# High-Quality Predicted Metabolite-Pathway Annotations Expand Pathway Coverage and Increase Enriched Pathway Detection Across Metabolomics Datasets

**DOI:** 10.3390/metabo16080545

**Published:** 2026-07-31

**Authors:** Erik D. Huckvale, P. Travis Thompson, Robert M. Flight, Hunter N. B. Moseley

**Affiliations:** 1 Markey Cancer Center, University of Kentucky, Lexington, KY 40506, USA; edhu227@uky.edu (E.D.H.); ptth222@uky.edu (P.T.T.); robert.flight@uky.edu (R.M.F.); 2 Superfund Research Center, University of Kentucky, Lexington, KY 40506, USA; 3 Department of Molecular and Cellular Biochemistry, University of Kentucky, Lexington, KY 40506, USA; 4 Institute for Biomedical Informatics, University of Kentucky, Lexington, KY 40506, USA

**Keywords:** annotation enrichment analysis, pathway enrichment analysis, metabolomics, metabolites, chemical structure, pathways, machine learning, graph convolutional neural network, multitask classification, extreme classification

## Abstract

**Background/Objectives**: Metabolism-level interpretation of metabolomics datasets requires aggregation analyses across metabolites. One highly used aggregation analysis is pathway enrichment analysis (PEA), which involves detecting pathways enriched with metabolites that are differential between experimental groups. Annotating metabolites with pathway associations is a prerequisite for PEA. While several knowledge bases define pathways and include metabolite-pathway annotations, these definitions are often partially or even grossly incomplete due to limitations in current metabolic knowledge and its curation, which greatly limits the effectiveness of PEA. **Methods**: In this work, we used our novel multitask classification, graph convolutional-like neural network to generate high-quality metabolite-pathway annotations for pathways defined across KEGG, MetaCyc, and Reactome. We then included these predicted metabolite-pathway annotations when performing PEA on 990 datasets deposited in Metabolomics Workbench. **Results**: We demonstrate an 8-fold increase in the median number of enriched pathways detected across these datasets compared to using only knowledge base-derived annotations. **Conclusions**: The significant increase in enriched pathways has the potential to substantially improve the biological and biomedical interpretability of metabolomics datasets.

## 1. Introduction

Metabolism refers to the interconnected network of chemical reactions within cells and organisms that sustain life processes. Metabolism is also directly involved in many biological and disease processes, so it is biologically and biomedically meaningful to detect and understand changes in metabolism. Since metabolites are reactants and products of chemical reactions in life processes [1,2,3], their systematic detection and characterization, i.e., metabolomics, provides many details about metabolism. Changes in metabolite abundances and fluxes between experimental groups can provide biological and biomedical insights, e.g., testing the effects of a drug, examining differences between diseased versus healthy groups, etc. [4,5].

However, it is very difficult to directly and systematically evaluate changes across hundreds to thousands of measured metabolites to generate biological and biomedical interpretations. To aid in systemic biological and biomedical interpretation of metabolomics datasets, data at the metabolite abundance and flux level can be aggregated to the pathway level, where a pathway can represent metabolic pathways, biological pathways, or any other collection of metabolites associated with some biological or biomedical concept. The metabolites that are part of such a collection are “associated with the pathway”. The most common pathway aggregation analysis of metabolomics datasets is pathway enrichment analysis (PEA), which is an annotation enrichment analysis (AEA) of pathway annotations, where each metabolite is annotated with the set of pathways it is associated with [6,7,8,9,10,11,12,13,14,15,16,17]. In PEA, evaluation metrics of metabolite abundance differences, like *p*-values, log-fold changes, etc., are analyzed to detect “enrichment” of metabolites associated with a given pathway. When the metabolites associated with a pathway collectively are enriched at a desired level of statistical significance (i.e., very low probability to be due to random chance), the pathway is defined as enriched.

In order to perform PEA, detected metabolites must have associated pathway annotations. Knowledge bases such as the Kyoto Encyclopedia of Genes and Genomes (KEGG) [18,19,20], MetaCyc [21], and Reactome [22] contain compound entries with pathway annotations, but only for some metabolites, and even then the metabolites with pathway annotations do not always have a complete set of annotations. Therefore, a recurring problem with PEA is a lack of pathway annotations available or accessible from such knowledge bases [11,23,24]. A main reason for this incompleteness is that the knowledge bases are at best based on “known” metabolism, if knowledge base curation is up-to-date with current peer-reviewed scientific literature. Both enzyme promiscuity and unknown (moonlighting) enzymatic functions represent significant portions of “unknown” metabolism that are still being discovered. This is evidenced by a significant fraction of compound entries in metabolic knowledge bases not being annotated to at least one pathway or enzymatic reaction. For example, over 60% of KEGG compound entries lack pathway annotations [25]. To further quantify these effects on incompleteness, we see that there are roughly 20,000 reactions in KEGG and MetaCyc combined, which is only 50% of the known enzymatic reactions in other knowledge bases like BRENDA, which has over 40,000 reactions [26]. Another reason is that different knowledge bases focus on different parts or aspects of metabolism [24]. Finally, many metabolites detected in metabolomics datasets do not easily correspond to the correct knowledge base entries without the database-specific compound identifier or some universal identifier like the IUPAC International Chemical Identifier (InChI) [27,28,29]. When performing PEA, missing pathway annotations greatly limit the number of enriched pathways detectable in metabolomics datasets, therefore limiting the biological and biomedical interpretability and insight gained from metabolomics datasets.

Solutions to reducing missing pathway annotations include both improving data curation to more comprehensively document the metabolite-pathway annotations that are known, as well as performing the wet lab research necessary to discover the metabolite-pathway associations that are still unknown. Currently, both tasks are prohibitively time-consuming and financially costly. In the future, data curation of known metabolism may be automated using deep learning models, as is being demonstrated in the generation of gene annotations [30,31]. However, there is no obvious approach for speeding up and reducing the cost of wet lab discovery of “unknown” metabolism. To more directly address the issue of missing pathway annotations, our lab is actively developing machine learning (ML) models for predicting the pathway associations of metabolites using chemical structures of metabolites as input [25,32,33,34,35,36,37]. Our current best approach, which is also the best demonstrated so far in the field [38], uses a graph convolutional-like multilayer perceptron (MLP) model that can predict metabolite-pathway annotations for a total of 22,265 pathway IDs defined across KEGG, MetaCyc, and Reactome, where 8517 of these are unique pathway definitions [38]. While a pathway ID is the label or name assigned to a pathway by its respective knowledge base, a pathway definition is the set of metabolites associated with the pathway. Since pathways of different IDs can have equivalent pathway definitions, the model can predict more pathway IDs than unique pathway definitions. In this work, we focus on the unique pathway definitions to avoid redundancy and overly optimistic performance results.

The single binary classification model uses paired metabolite and pathway atom coloring features [39,40,41] representing all 1-bond, 2-bond, and 3-bond subgraphs in the metabolite and across metabolites annotated to a pathway. This enumeration of all chemical subgraphs provides similar intermediate results to a graph convolutional neural network [42,43], with the resulting features providing a representation of the chemical graph that can be used for global graph classification tasks. The pairing of metabolite-specific and pathway-specific chemical subgraph features creates a multitask classification model [44,45,46] and, more specifically, a multitask extreme classification model [36,47,48], enabling transfer learning [49,50,51] between specific pathway prediction tasks [34,35,37,38]. Huckvale and Moseley demonstrated model performance with a mean Matthew’s Correlation Coefficient (MCC) [52] of 0.9036 ± 0.0033 (sd), computed by 100 90%:10% train-test splits for a dataset with roughly 50,000,000 feature vectors [38]. Also, the model became significantly more generalizable when using InChI standardization [27,28,29] of the chemical structure representations prior to training, testing, and novel prediction [38]. The main limitation in the model is poorer prediction performance on novel pathways not used in the training of the model, which is minor considering the model was trained with over 8000 unique pathway definitions. In this work, we demonstrate how high-quality predicted metabolite-pathway annotations generated from this model increase the median number of enriched unique pathway definitions 8-fold across a large analysis that reuses 990 metabolomics datasets obtained from the Metabolomics Workbench (MW) repository [53]. We fully expect the increased number of enriched pathways, detectable as a result of the increased pathway annotations, will substantially improve the results of future metabolomics PEA and meaningfully improve the biological and biomedical interpretability and insight obtainable in the field of metabolomics.

## 2. Materials and Methods

### 2.1. Downloading, Cleaning, and Filtering Metabolomics Datasets

To analyze the improvement in PEA afforded by our ML model’s pathway annotation predictions, we used the mwtab Python package [54,55], previously developed and recently improved in our lab, to download 4372 metabolomics datasets in mwTab format from the Metabolomics Workbench (MW) repository [53] on 26 April 2024. We then used a prototype rcha_metab (repair, clean, harmonize, augment) Python package, being developed in our lab but not yet publicly available, to repair unparsable mwTab files, clean the resulting files, and harmonize field names and values, in order to make them usable in our meta-analyses. Due to the large number of formatting and harmonization issues in MW deposited datasets [55,56], the vast majority of the downloaded datasets would not have been usable in the PEA meta-analyses presented here without the application of the prototype rcha_metab package.

**Figure 1:**
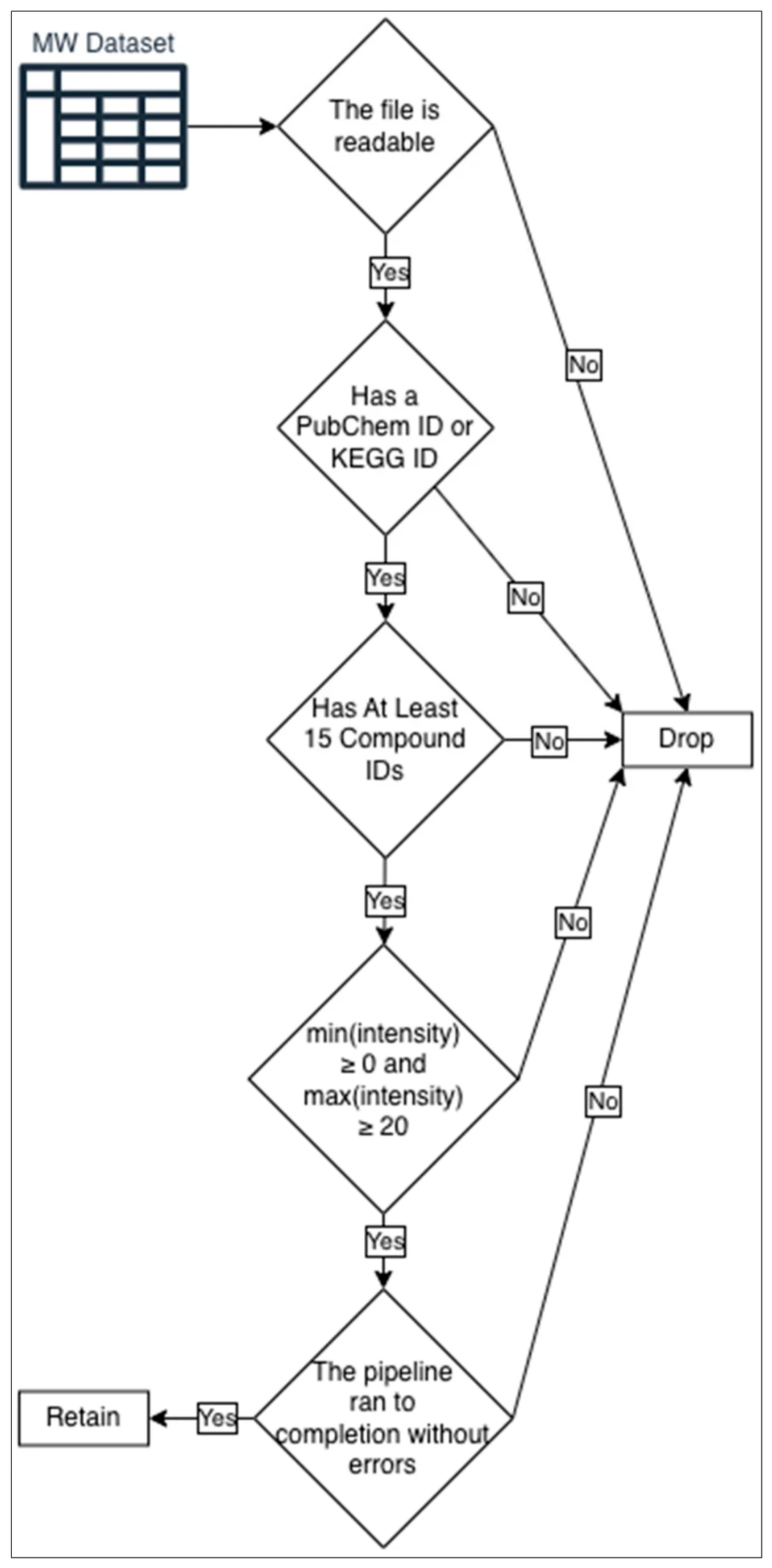
Checks made to determine whether to filter or keep an MW dataset for the analysis.

Connecting metabolites in the MW datasets to compound identifiers (IDs) in a major metabolic or chemical knowledge base like KEGG and PubChem [57,58] is a crucial step to detecting enriched pathways. Since the chemical structure information is not provided with the MW data, the compound ID must be known to retrieve the corresponding molfile [59] containing the chemical structure data needed by the ML model to predict pathway annotations. As our analysis involved comparing the effects of the known annotations to the predicted annotations, we also needed the compound IDs for at least some of the metabolites to access the known pathway annotations from KEGG, MetaCyc, and Reactome. While the KEGG, MetaCyc, and Reactome (ChEBI) compound IDs enabled retrieving both a molfile and known pathway annotations, when only a PubChem ID was available, we could only use the predictions. Very few MW datasets have compound IDs for MetaCyc or ChEBI [60] (and thus to Reactome), but IDs from these knowledge bases can be retrieved from KEGG or PubChem IDs via cross-referencing. This meant we at least needed either a KEGG ID or a PubChem ID, and this requirement significantly reduced the number of usable datasets from the MW repository. Another requirement not met by all MW datasets was needing a sufficient number of compound IDs, even if some were available. For a pathway to be reasonably detectable as enriched at an adjusted *p*-value ≤ 0.01, it must be associated with at least 15 metabolites. In some cases, the MW dataset did not contain at least 15 metabolites at all, regardless of whether their IDs were provided or not. Thus, if there were fewer than 15 compound IDs in the dataset, it is pragmatically impossible for any pathways to be detectable, and we filtered out the dataset. Other relatively minor requirements not met included the metabolite intensities being at an appropriate scale (i.e., the minimum intensity being greater than or equal to 0 and the maximum intensity being greater than or equal to 20), the file being readable by the mwtab Python package, and the data processing pipeline being capable of finishing without software errors. Figure 1 details the checks made to determine whether to keep or filter an MW dataset in the order that they were checked. Table S1 details the number of MW datasets filtered per requirement not met, totaling 3382 datasets filtered out, reducing the number of MW datasets usable for our analysis from 4372 to 990.

### 2.2. Resolving PubChem SIDs and CIDs

While PubChem does not contain pathway annotations [57,58], the annotations of pathways defined in the KEGG, MetaCyc, or Reactome knowledge bases can be obtained by cross-referencing the PubChem ID to a compound ID in one of these three knowledge bases, as well as by using the ML model on the provided PubChem molfiles [59] to predict pathway annotations. However, one specific issue that came up when using PubChem IDs was that PubChem has both CIDs (compound IDs) and SIDs (substance IDs) [57,58]. This is a problem because both sets of IDs are just positive integers; they overlap, and most MW datasets do not indicate whether they are using CIDs or SIDs. Therefore, when given an arbitrary PubChem ID, it could be either a CID or an SID. Since PubChem provides metabolite cross-references to KEGG, MetaCyc, and Reactome (ChEBI) via SIDs, and the file names of their molfiles contain CIDs, it must be known whether a PubChem ID in an MW file is an SID or a CID. This required us to write a Python script to compare the metadata of the metabolites in the MW datasets to both CID and SID records in PubChem to determine the correct type of PubChem ID. The script first compared the synonyms or names for the metabolite with the name given in the MW dataset. If a name strongly matched a CID record, we recorded the PubChem ID as a CID; otherwise, it was recorded as an SID. If the name was neither matched to a CID record nor an SID record, then the chemical formula and molecular mass, if available in the metadata, were compared to make a match. Molecular mass and chemical formula matching were simple equality comparisons, but the name matching drew on code developed for the rcha_metab package. The name comparisons included Levenshtein distance matching (fuzzy matching using the fuzzywuzzy Python package (v0.18.0) [61]), and various other matching techniques based on specific naming conventions in different sub-classifications of metabolites, such as lipids, isotopologues, and isomers. If a PubChem ID neither matched to an SID nor CID due to limitations or inconsistencies in the metadata (e.g., the ID corresponded to a CID while the synonyms in fact matched an SID), then the PubChem ID could not be used.

### 2.3. Chaining Compound ID Cross-References

We used the web-based cross-referencing functionality of PubChem to obtain compound IDs for KEGG, MetaCyc, and ChEBI/Reactome, converting PubChem CIDs to PubChem SIDs as needed. PubChem provides cross-references via its REST API mapping SIDs to external IDs in KEGG, MetaCyc, and ChEBI. If a metabolite in an MW dataset has an SID available, we can use the SID to map directly to cross-references. However, if a CID is available, we first must map to its corresponding SIDs. Iterating through a list of SIDs corresponding to a given CID, we selected the cross-references of the first SID in the list with a cross-reference available, if any. We did this for KEGG, MetaCyc, and ChEBI cross-references for each metabolite with either an SID or CID available. Likewise, we used the web-based cross-referencing functionality available in KEGG, MetaCyc, and ChEBI to map compound IDs back to PubChem and each other. Finally, we implemented a Python script to perform chained cross-referencing, i.e., cross-referencing back-and-forth between all four knowledge bases and seeing if the number of available metabolite IDs increased. The Python script repeats the back-and-forth cross-referencing, further augmenting the number of PubChem, KEGG, MetaCyc, and ChEBI IDs in the MW dataset until there is no further increase in compound IDs.

### 2.4. Accessing Known Metabolite-Pathway Annotations

We used the KEGG IDs directly available in an MW dataset to access known metabolite-pathway annotations in KEGG. We refer to these known metabolite-pathway annotations as the “ground truth”, which is information known to be true but is not expected to be complete. We then used the KEGG, MetaCyc, and ChEBI compound IDs generated from the chained cross-referencing or already present in the MW dataset to access known metabolite-pathway annotations in KEGG, MetaCyc, and Reactome. We refer to these known metabolite-pathway annotations as the “ground truth with cross-referencing”. As of 19 November 2024, there are 23,110 pathway IDs available across these three knowledge bases. However, many of these pathway IDs have redundant pathway definitions, i.e., the set of metabolites associated with the pathway. While there are 23,110 pathway IDs, 8574 of these pathways have unique pathway definitions (Table 1).

### 2.5. Predicting Metabolite-Pathway Annotations

**Figure 2:**
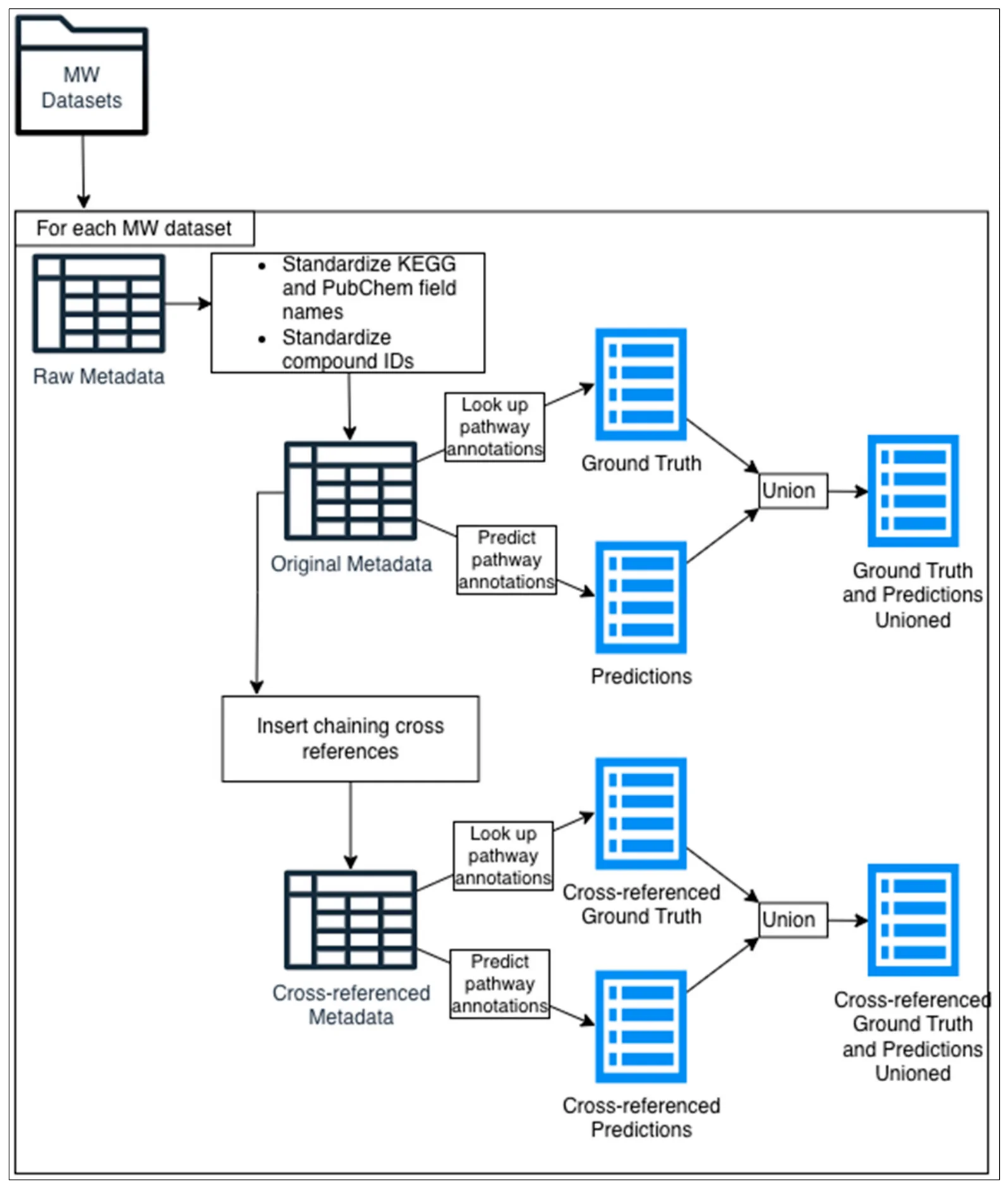
Using the MW datasets’ metadata to determine compound IDs and map them to pathway annotations.

We used the extreme classification model described in Huckvale and Moseley [38] to generate predicted metabolite-pathway annotations. Hyperparameters for this model are in Table S5. This single binary classification model used a prediction threshold of 0.678975. All predicted metabolite-pathway annotations were retained from the model. More specifically, we used the KEGG compound IDs available in an MW dataset to predict pathway annotations for KEGG, MetaCyc, and Reactome using the molfiles provided by KEGG and standardized using InChI canonicalization. Next, we used the ML model to predict annotations to the pathways defined in KEGG, MetaCyc, and Reactome using the InChI-standardized molfiles from the PubChem CID records for all PubChem CIDs available in the MW datasets, converting PubChem SIDs to PubChem CIDs as needed. PubChem molfiles are only available by their corresponding CID. If a CID is available for a metabolite in an MW dataset, we obtained the molfile directly. If an SID was available, we mapped the SID to its corresponding CID. We refer to these metabolite-pathway annotations obtained by predicting on chemical structure information from the molfiles as the “predicted annotations”. Next, we used the KEGG, MetaCyc, ChEBI, and PubChem compound IDs generated from the chained cross-referencing or already present in the MW dataset to access molfiles in the respective knowledge base, which we standardized using InChI canonicalization, and then used to predict metabolite-pathway annotations for KEGG, MetaCyc, and Reactome-defined pathways. We refer to these predicted metabolite-pathway annotations as the “predicted annotations with cross-referencing”. Figure 2 details the process of obtaining pathway annotations, including looking up known pathway annotations and predicting additional pathway annotations for both the original and cross-referenced datasets. While there were molfiles used by the ML model to predict pathway annotations for the respective metabolites, some of the molfiles used for constructing the dataset to train the model could not be successfully standardized. Because of this, some of the pathways in the dataset lost associated metabolites in their definitions. And some of these pathways lost all associated metabolites, resulting in those pathways being removed from the dataset. The ML model can only predict pathways available in the dataset it was trained on. Therefore, while 23,110 pathway IDs and 8574 pathway definitions are known and available for the ground truth, 22,265 pathway IDs and 8517 unique pathway definitions remained predictable by the ML model (Table 1).

### 2.6. Pathway Enrichment Analysis

As the increase in enriched pathways could be attributed to the increase in available cross-references, to prove the utility of the ML model beyond our chained cross-referencing algorithm, we computed enriched pathways both on the cross-referenced version of the MW datasets and the original dataset. We also compared the impact on the number of enriched pathways using the ground truth alone, the predictions alone, and the ground truth unioned with the predictions.

For all combinations of the original and cross-referenced MW datasets with ground truth annotations, predicted annotations, and ground truth unioned with predictions, we used the categoryCompare2 R package [62,63] developed in our lab to compute the number of enriched pathways for all 990 usable MW datasets. Specifically, we performed PEA using the Gene Set Enrichment Analysis (GSEA) [64] algorithm implemented in fgsea (v1.32.4) [65] and wrapped by categoryCompare2 (v0.200.5). GSEA requires the features (metabolites in our case) to be ranked. We performed Principal Component Analysis (PCA) [66] on the log-transformed metabolite intensity values in the MW datasets. To prevent values of negative infinity resulting from a log of 0, we replaced 0s with the minimum non-zero value divided by 10 prior to the log transform. Prior to the log transform, we centered the metabolite abundances in each sample by dividing by the median intensity across metabolites in that sample (sample median normalization). Next, we used the resulting principal component (PC) loadings as the ranks for GSEA. Only the top PCs adding up to at least 75% of the total variance in the given dataset were used. With the categoryCompare2 package, we performed GSEA on each list of PC loadings followed by Benjamini–Hochberg multiple testing correction [67]. Pathways with an adjusted *p*-value ≤ 0.01 were considered enriched. This approach obviates the need to determine the experimental design of a given metabolomics dataset, since differences between experimental groups (i.e., systematic variance) are expected to be represented by the top PCs. However, other sources of variance such as analytical variance and batch effects will also be detected as differences in certain PCs. But this does not affect the overall purpose of evaluating effects of predicted metabolite-pathway annotations on pathway enrichment analysis. Also, it is possible to detect an enriched pathway in multiple PCs; however, in this context, each PC is treated as a separate pathway enrichment analysis. Furthermore, this approach enables the analysis of all 990 metabolomics datasets to be fully automated. Parameters for GSEA included only detecting pathways with a minimum of 15 metabolites and a maximum of 500 metabolites. As GSEA uses stochastic permutation, a random seed was selected from the current process ID.

**Figure 3:**
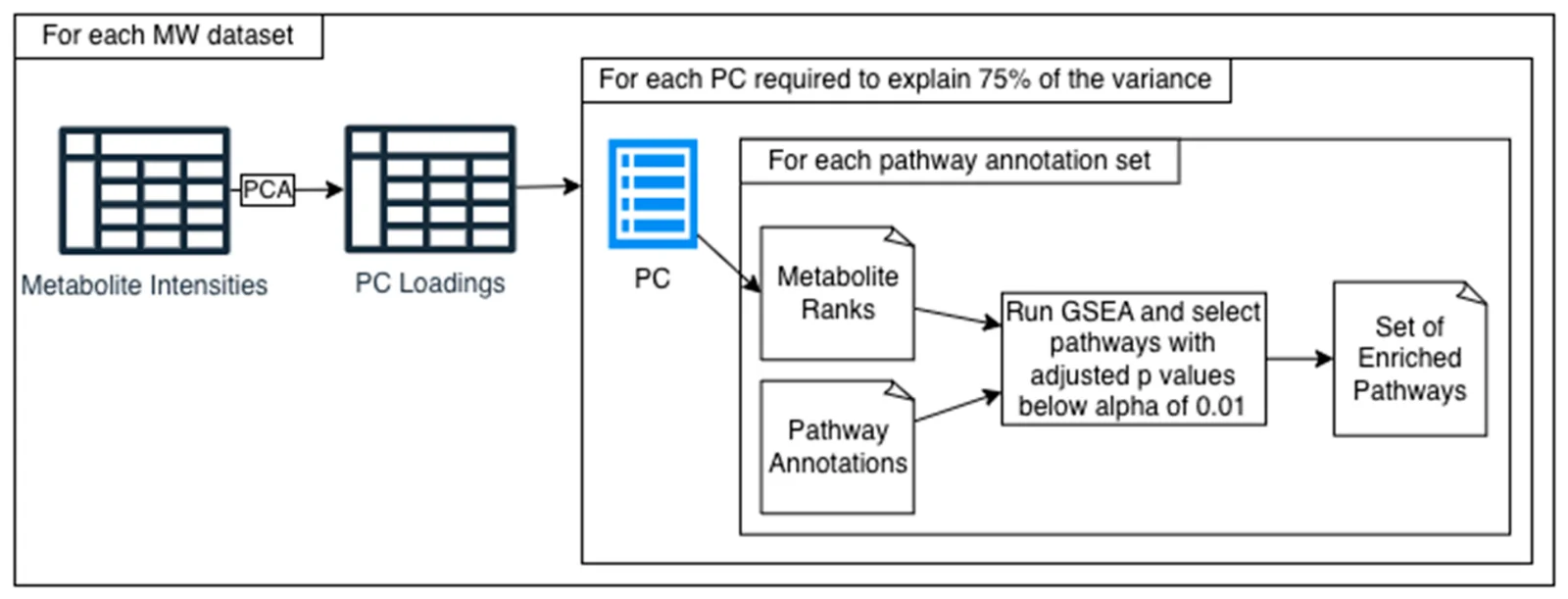
Detecting enriched pathways for each combination of PC and pathway annotation set.

Finally, we statistically compared the number of enriched pathways between the original and cross-referenced datasets as well as between the datasets using ground truth annotations, predictions, and ground truth unioned with predictions using Mann–Whitney U tests [68] and Wilcoxon signed-rank tests [69]. While the matched-pair case–control experimental design allows the use of a Wilcoxon signed-rank test, we conservatively calculate the Mann–Whitney U test as well, since the dataset had ample sample size for high statistical power. We do these same statistical tests comparing information gain, i.e., pathways enriched only when using predictions, and information loss, i.e., pathways enriched only when using ground truth. Figure 3 details the process of detecting enriched pathways. Note that when counting the number of detected pathways for a given set of pathway annotations for a given MW dataset, the counts are summed across the PCs selected to explain 75% of the variance. The summation of enriched pathways across PCs is not double-counting because each PC is an orthogonal component of correlated variance, which is separately being evaluated for improvement in statistical sensitivity and power.

To compare the number of pathways detected based on the predictions from our model to pathways predicted by random chance (i.e., a negative control), we created a random prediction model. This model assigns a metabolite as being involved in a pathway randomly, with the probability, *p*, of being assigned equal to the number of compounds assigned to that pathway divided by the total number of entries with that pathway in the training set, i.e., *p* = number of positive entries/(number of positive entries + number of negative entries). Therefore, the probability of a metabolite not being assigned was equal to 1 − *p*. This metric ensures that metabolites are more likely to be assigned to larger pathways and the proportion of positive predictions is close to the proportion of positive entries in the training set. We evaluated the model’s performance by running 100 CV iterations. Then we used this random prediction model to make predictions for the metabolites in the MW datasets just as we did with the MLP. Using these predictions, we determined the number of pathways enriched when using the random predictions as compared to using the MLP’s predictions.

### 2.7. Description of Software Packages and Hardware Used

While the categoryCompare2 (v0.200.5) package [62,63], used to perform PEA via the GSEA algorithm, is written in the R programming language (v4.4.1) [70], all other code was written in the Python (v3.10.12) programming language [71], including the mwtab (commit SHA 38f43e3f719d456a98c888095b539922eef5fe27 between v1.2.5 and v2.0.0) package [54,55]. The basic data processing was performed using the Pandas (v2.2.3) [72] and NumPy (1.26.4) [73] Python packages. PCA was performed using the Scikit-learn (v1.3.0) Python package [74]. The Mann–Whitney U tests and Wilcoxon signed rank tests were performed using the SciPy (v1.16.3) [75] Python package. We generated all of the data visualizations of results using the Tableau Public business intelligence software (v2024.3.3) [76] as well as the seaborn (0.12.2) Python package [77] built on top of the Matplotlib package (v3.7.2) [78] within Jupyter Notebooks (v1.1.1) [79].

The hardware for this work included computers with 64 gigabytes (GB) of random-access memory (RAM) and central processing units (CPUs) ranging from 3.4 gigahertz (GHz) to 3.6 GHz, each with 6 hyperthreaded (HT) cores. The CPU chips included ‘Intel(R) Core(TM) i7-2600 CPU@3.40 GHz’, ‘Intel(R) Core(TM) i7-5930K CPU@3.50 GHz’, ‘Intel(R) Core(TM) i7-4930K CPU@3.40 GHz’, and ‘Intel(R) Core(TM) i7-6850K CPU@3.60 GHz’.

## 3. Results

### 3.1. Pre-GSEA

**Figure 4:**
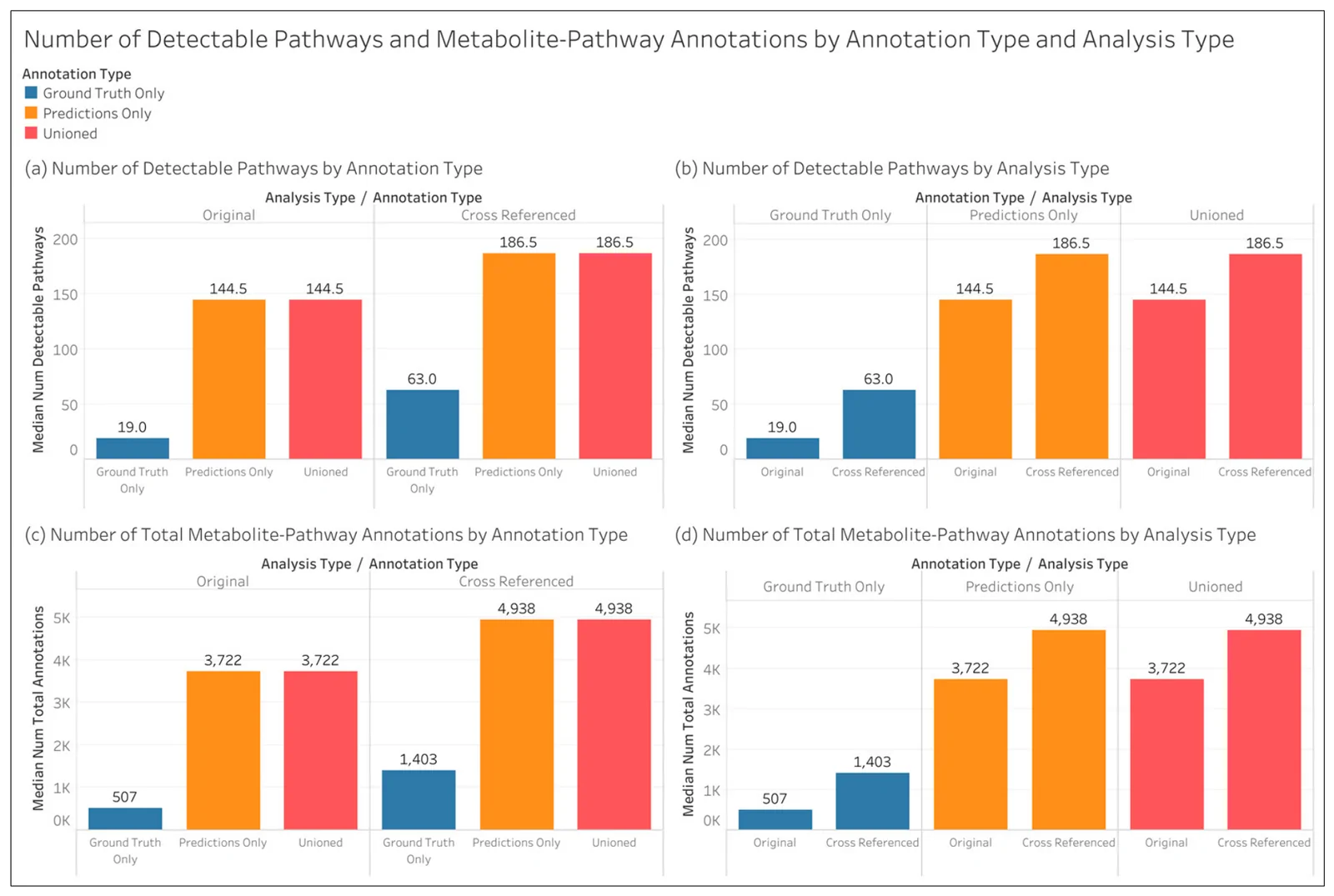
The median across MW datasets of the number of pathways that are detectable (associated with at least 15 metabolites) and the total number of metabolite-pathway associations. Results are for unique pathway definitions. The annotation types are defined as follows: Ground Truth Only: Known pathway annotations as defined in knowledge bases; Predictions Only: Predicted pathway annotations alone; Ground Truth and Predictions Unioned: The unioned set of ground truth pathway annotations and predicted pathway annotations. The analysis types are defined as follows: Original: Derived from MW datasets containing the knowledge base compound IDs as provided by the original metadata; Cross-referenced: Derived from MW datasets containing the larger set of knowledge base compound IDs as a result of chained cross-referencing.

The following results describe the annotations prior to GSEA, where the pathways counted are unique pathway definitions rather than pathway IDs. For a pathway to be detectable, the given MW dataset must have at least 15 metabolites with pathway annotations to the specific pathway. Figure 4a shows how the median number of detectable pathways across MW datasets changes depending on the set of metabolite-pathway annotations used. We see that using the ML model’s predictions greatly increases the number of pathways that are detectable. We also see that the median does not change whether using the Predictions Only or using the Predictions Unioned with Ground Truth. Figure 4b shows the same quantities as Figure 4a but rearranged to compare how adding metabolite ID cross-references impacts the median number of detectable pathways. We see that regardless of the annotations we use, the cross-referenced datasets have a meaningfully higher number of detectable pathways. Figure 4c shows the median of the total number of metabolite-pathway annotations across MW datasets, i.e., the sum of all metabolite associations across all pathways in a dataset. Likewise, adding predictions greatly increases the median number of metabolite-pathway annotations, and Figure 4d shows that adding cross-references does the same; however, the increase from predictions is substantially larger. Figure S1 shows these results as violin plots to illustrate the distribution of the number of detectable pathways and total number of metabolite-pathway annotations across the datasets. While Figure 4 shows these results for unique pathway definitions, Figure S2 shows these results for individual pathway IDs. And while these are the results of KEGG, MetaCyc, and Reactome combined, Figure S3 shows this information for each knowledge base separately (unique pathway definitions). The largest increases occur for MetaCyc- and Reactome-defined pathways, but these two knowledge bases represent over 90% of the uniquely defined pathways. Figure S4 shows these per knowledge base results for individual pathway IDs.

**Figure 5:**
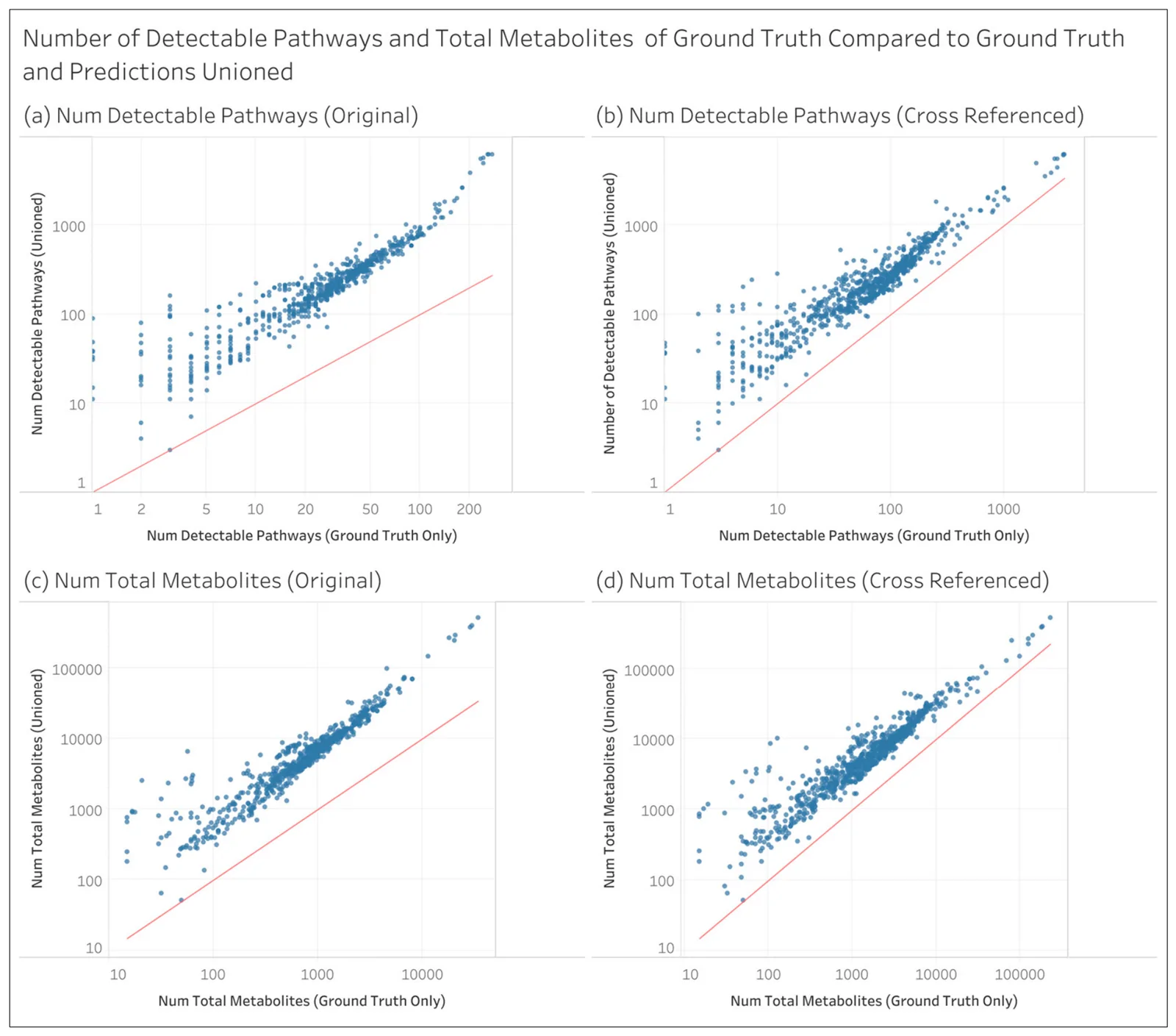
Per-dataset view of the amount of increase in the number of detectable pathways and the total number of metabolite-pathway associations between ground truth annotations and the ground truth unioned with the predictions. Note that the axes are on different log scales across the 4 scatterplots. Each point is a separate MW dataset. Results are for unique pathway definitions. The annotation types are defined as follows: Ground Truth Only: Known pathway annotations as defined in knowledge bases; Ground Truth and Predictions Unioned: The unioned set of ground truth pathway annotations and predicted pathway annotations. The analysis types are defined as follows: Original: Derived from MW datasets containing the knowledge base compound IDs as provided by the original metadata; Cross-referenced: Derived from MW datasets containing the larger set of knowledge base compound IDs as a result of chained cross-referencing. The red diagonal line has a y = x relationship.

Figure 5 shows, on a per MW dataset basis, the change in detectable pathways and metabolite-pathway associations between ground truth and the ground truth unioned with predictions on log-log scatterplots. The red line in each scatterplot represents y = x with a slope of 1. We see that whether using the original or the cross-referenced compound IDs, while there are a few MW datasets that experienced little or no change, nearly all datasets experienced a meaningful increase in both the number of detectable pathways and metabolite-pathway associations. Figure S5 shows these results for individual pathway IDs. Figures S6–S9 show this information for each knowledge base separately. Again, the largest increases occur for MetaCyc-defined and Reactome-defined pathways, which represent over 90% of the uniquely defined pathways. Figures S10–S13 show the same as Figures S6–S9 but for individual pathway IDs.

### 3.2. Post-GSEA

While the above results demonstrate how the ML model’s predictions increase the number of pathways that could be detected, the following results demonstrate the actual change in enriched pathways at an adjusted *p*-value ≤ 0.01. Again, these results are for unique pathway definitions where a pathway definition is the set of metabolites known to be associated with it as defined in a pathway’s respective knowledge base. This is distinct from pathway IDs, which are names given to pathways in their knowledge bases, but multiple pathways of different IDs may have the same pathway definition.

#### 3.2.1. Changes in the Number of Enriched Pathways

**Figure 6:**
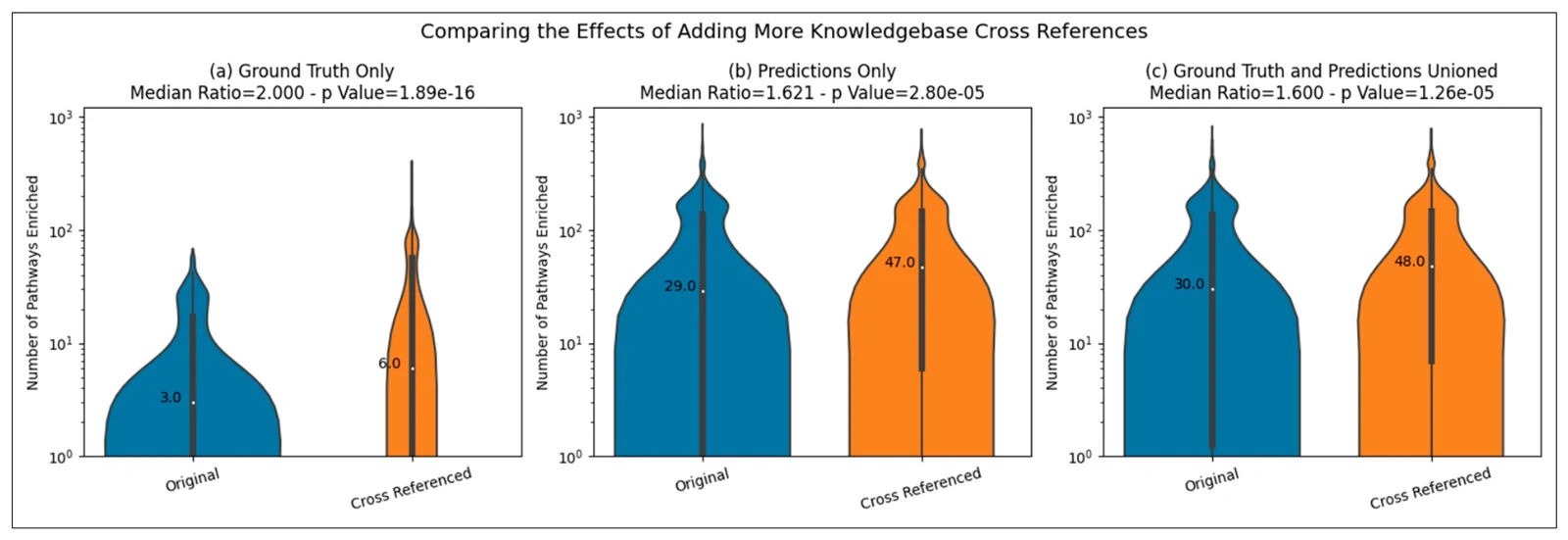
The effect on the number of enriched pathways of adding metabolite ID cross-references to the MW datasets for each type of annotation. We see a moderate increase in the number of enriched pathways when incorporating cross-references. Results are for unique pathway definitions. The annotation types are defined as follows: Ground Truth Only: Known pathway annotations as defined in knowledge bases; Predictions Only: Predicted pathway annotations alone; Ground Truth and Predictions Unioned: The unioned set of ground truth pathway annotations and predicted pathway annotations. The analysis types are defined as follows: Original: Derived from MW datasets containing the knowledge base compound IDs as provided by the original metadata; Cross-referenced: Derived from MW datasets containing the larger set of knowledge base compound IDs as a result of chained cross-referencing. Analyses with 0 enriched pathways for both ground truth and predictions are filtered from the groups being compared. The sizes of the groups are as follows: (**a**) 770, (**b**) 770, (**c**) 770.

Figure 6 shows the change in the median number of enriched pathways across MW datasets after adding more compound IDs via cross-referencing. Datasets are excluded if they lack enriched pathways in both groups being compared. We see that adding the cross-references moderately increases the median number of enriched pathways. Figure S14 shows these results for individual pathway IDs. Figure 6 shows *p*-values from a Mann–Whitney U test. The *p*-values for the Wilcoxon signed rank test are as follows: (a) 1.09 × 10^−76^, (b) 3.08 × 10^−25^, (c) 1.18 × 10^−24^.

**Figure 7:**
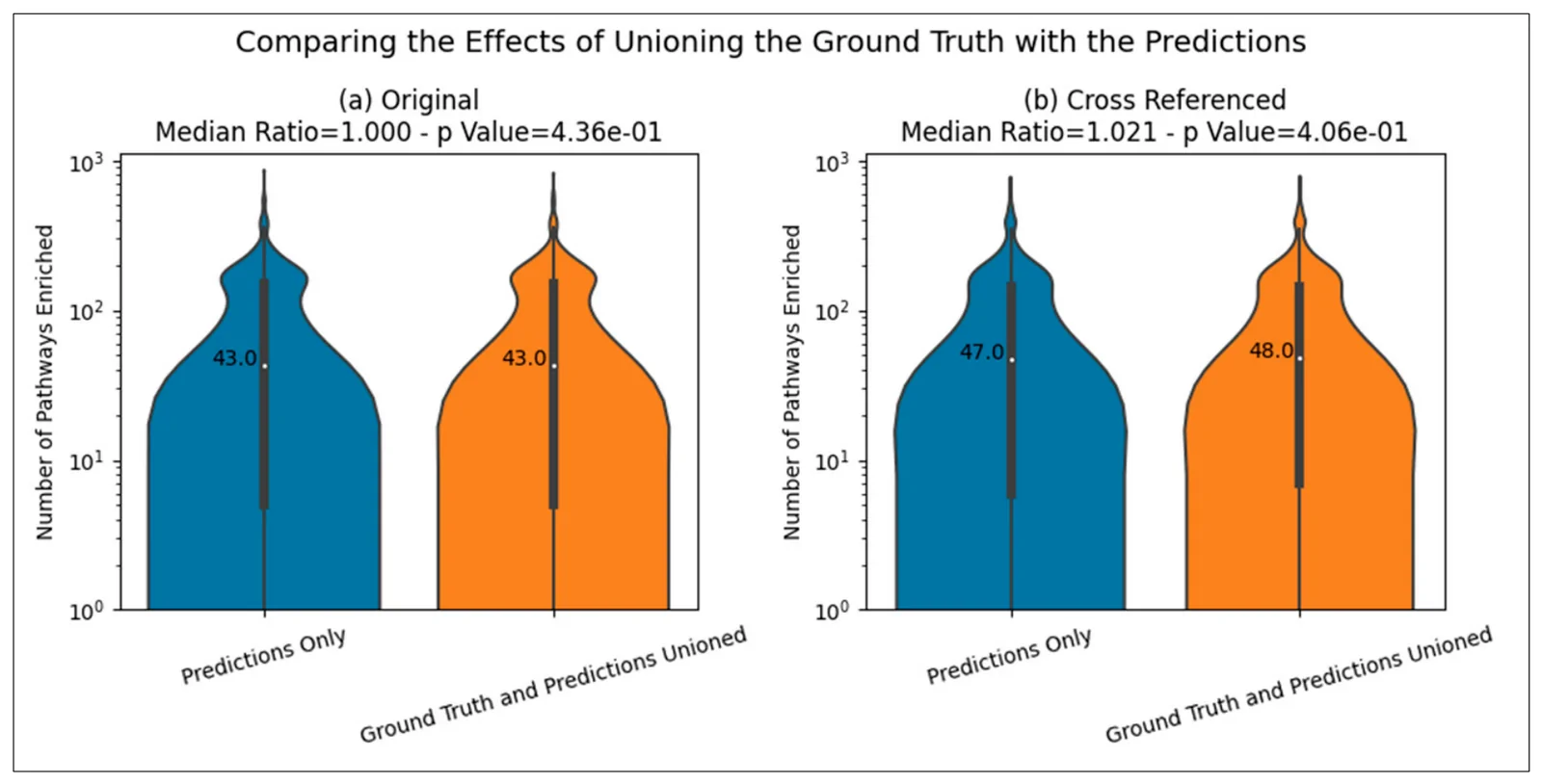
The effect on the number of enriched pathways when unioning the predictions with the ground truth. We observe no significant difference in the number of pathways enriched when using either predictions by themselves or Predictions Unioned with Ground Truth. Results are for unique pathway definitions. The annotation types are defined as follows: Predictions Only: Predicted pathway annotations alone; Ground Truth and Predictions Unioned: The unioned set of ground truth pathway annotations and predicted pathway annotations. The analysis types are defined as follows: Original: Derived from MW datasets containing the knowledge base compound IDs as provided by the original metadata; Cross-referenced: Derived from MW datasets containing the larger set of knowledge base compound IDs as a result of chained cross-referencing. Analyses with 0 enriched pathways for both ground truth and predictions are filtered from the groups being compared. The sizes of the groups are as follows: (**a**) 689, (**b**) 770.

Figure 7 compares the number of enriched pathways when using the predictions by themselves and when unioning the ground truth with the predictions. We see that for both the original and cross-referenced MW datasets, there is not a significant difference between the two approaches. Figure S15 shows these results for individual pathway IDs. The *p*-values in Figure 7 are from a Mann–Whitney U test. The *p*-values from the Wilcoxon signed rank test are as follows: (a) 2.35 × 10^−1^ (b) 6.45 × 10^−2^.

**Figure 8:**
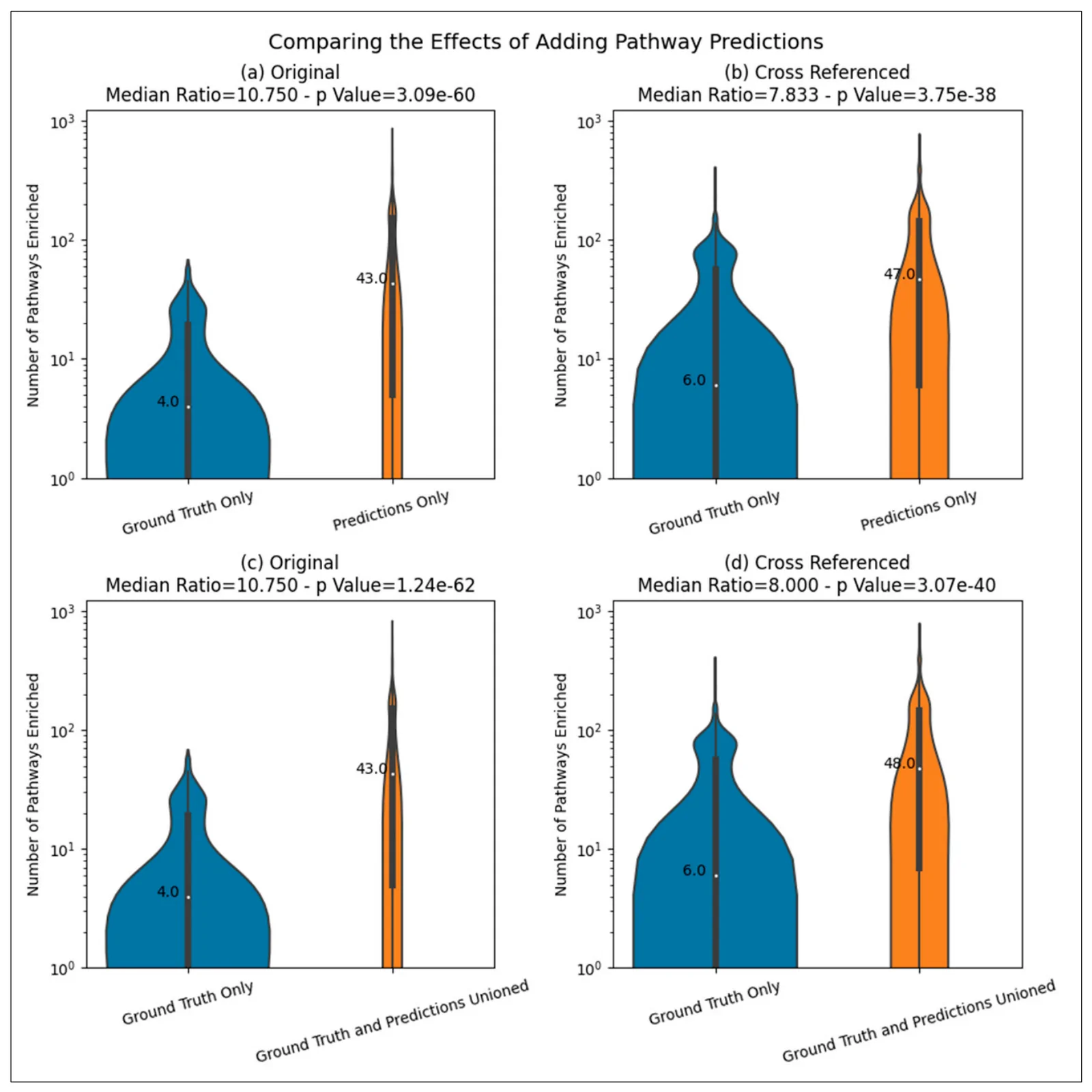
The increase in the number of enriched pathways when adding predicted annotations. In all combinations, we see a significant increase in the number of enriched pathways when incorporating pathways predicted by the ML model. Results are for unique pathway definitions. The annotation types are defined as follows: Ground Truth Only: Known pathway annotations as defined in knowledge bases; Predictions Only: Predicted pathway annotations alone; Ground Truth and Predictions Unioned: The unioned set of ground truth pathway annotations and predicted pathway annotations. The analysis types are defined as follows: Original: Derived from MW datasets containing the knowledge base compound IDs as provided by the original metadata; Cross-referenced: Derived from MW datasets containing the larger set of knowledge base compound IDs as a result of chained cross-referencing. Analyses with 0 enriched pathways for both ground truth and predictions are filtered from the groups being compared. The sizes of the groups are as follows: (**a**) 689, (**b**) 770, (**c**) 689, (**d**) 770.

Figure 8 demonstrates the high quality of the ML model’s predictions by the substantial increase in the median number of enriched pathways detected at an adjusted *p*-value ≤ 0.01 when adding the predicted pathway annotations. Although the effect size when comparing the original datasets to the datasets with predictions (10.75 for both Predictions Only and Predictions Unioned with Ground Truth) is larger than the effect size when comparing the cross-referenced datasets with predictions (7.833 for Predictions Only and 8.0 for Predictions Unioned with Ground Truth), all effect sizes still have an over 7-fold increase in the median number of enriched pathways after adding predicted pathway annotations. Figure S16 shows these results for individual pathway IDs, wherein we see an over 10-fold increase. Figures S17 and S18 show this information for each knowledge base separately. Again, the largest increases were observed for MetaCyc and Reactome-defined pathways, which represent over 90% of the uniquely defined pathways. Figures S19 and S20 show the same results as Figures S17 and S18 but for individual pathway IDs. The *p*-values in Figure 8 are from a Mann–Whitney U test. The *p*-values from the Wilcoxon signed rank tests are as follows: (a) 9.98 × 10^−98^, (b) 6.32 × 10^−102^, (c) 7.16 × 10^−99^, (d) 4.63 × 10^−103^.

#### 3.2.2. Information Loss and Gain

**Figure 9:**
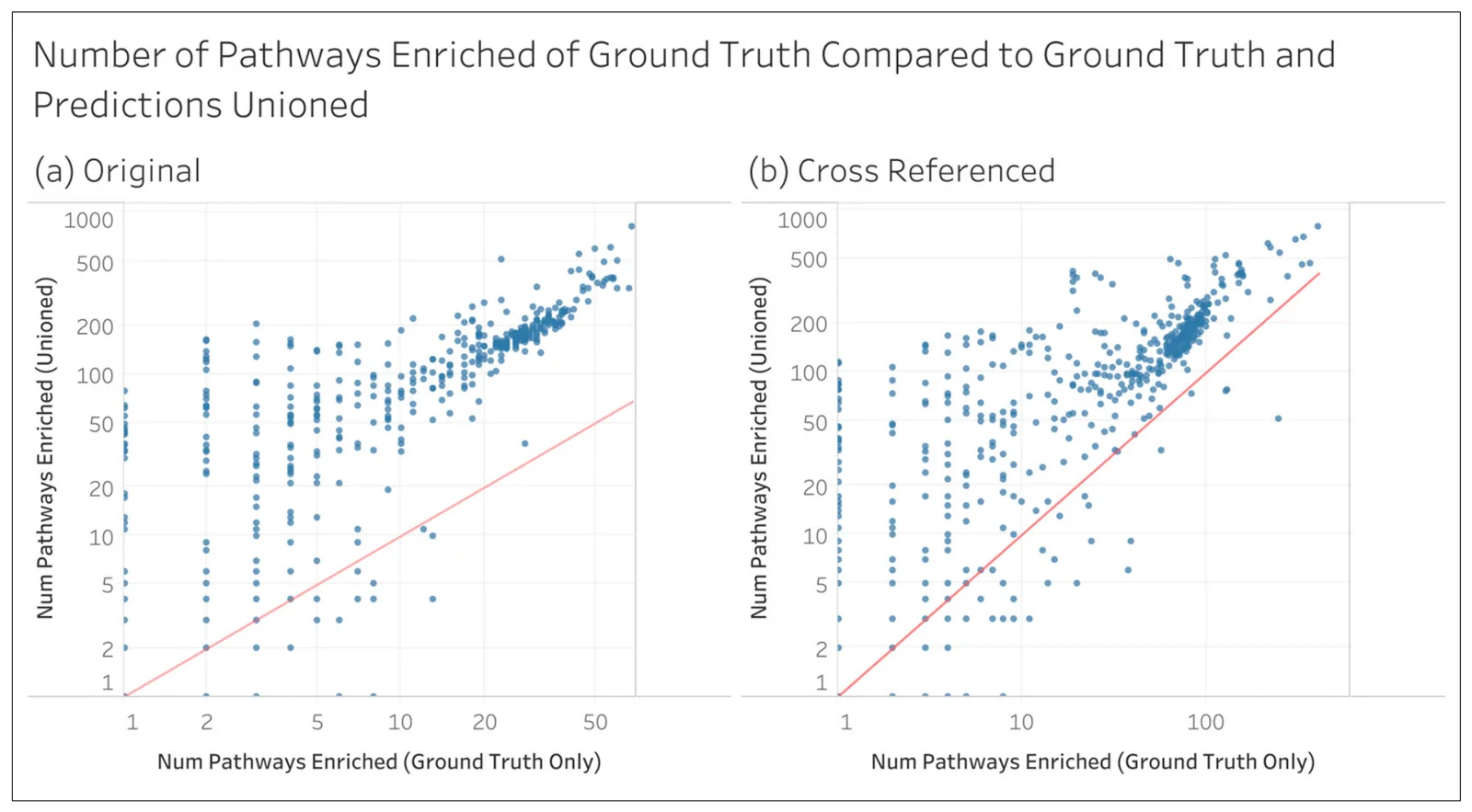
The per-dataset change in the number of enriched pathways between the ground truth and the ground truth unioned with the predictions. Each point is a separate MW dataset. We see that most datasets experience an increase in the number of enriched pathways after incorporating predicted pathways, while a few experience less enrichment after doing so. Results are for unique pathway definitions. The annotation types are defined as follows: Ground Truth Only: Known pathway annotations as defined in knowledge bases; Ground Truth and Predictions Unioned: The unioned set of ground truth pathway annotations and predicted pathway annotations. The analysis types are defined as follows: Original: Derived from MW datasets containing the knowledge base compound IDs as provided by the original metadata; Cross-referenced: Derived from MW datasets containing the larger set of knowledge base compound IDs as a result of chained cross-referencing. The number of analyses displayed is as follows: (**a**) 689 (**b**) 770. The red diagonal line has a y = x relationship.

While the above results demonstrate that adding the predictions greatly increases the overall number of enriched pathways, in some cases, incorporating the pathway predictions results in a relatively minor decrease in the number of enriched pathways. Figure 9 shows the per-MW dataset changes in the number of enriched pathways between the ground truth and that unioned with predictions as log-log scatterplots. Datasets with a non-zero number of pathways enriched for both ground truth and predictions are shown. The red line is a slope of 1, meaning data points above the line indicate an increase in enriched pathways after adding predictions and those below the line indicate a decrease. Consistent with prior results, we see that a majority of MW datasets experienced an increase in the number of enriched pathways as a result of adding predictions (71.3% to 89.9%), but for some datasets, adding the predictions actually resulted in fewer enriched pathways than when using the ground truth alone (1.3% to 4.5%). Figure S21 shows these results for individual pathway IDs. Figures S22 and S23 show this information for each knowledge base separately. Again, the largest increases were observed for MetaCyc and Reactome-defined pathways, which represent over 90% of the uniquely defined pathways. Figures S24 and S25 show the same results as Figures S22 and S23 but for individual pathway IDs.

Table 2 quantifies the information gain and information loss in terms of enriched pathways across MW datasets. Datasets with 0 enriched pathways for both ground truth and predictions are filtered out. Information loss is indicated when a pathway is enriched in the ground truth but no longer enriched after adding the predictions for a given MW dataset. Likewise, information gain is indicated when a pathway is not enriched in the ground truth but is enriched after adding the predictions for a given MW dataset. There is no change in information when a pathway is enriched from both sets of annotations for a given MW dataset. Total information loss, total information gain, and total unchanged information are summed up across all pathways across the MW datasets. Adding up these three sums provides the total number of enriched pathways across the MW datasets. The total can then be used to calculate the percentage of information loss, information gain, and unchanged information. Table 2 shows that whether using the original MW datasets or the cross-referenced datasets and whether using the Predictions Only or those unioned with ground truth, information gain has the highest percentage by far, with the percent unchanged coming in second. However, we do observe a small amount of information loss across MW datasets as a result of adding the predictions. Table S2 shows these results for individual pathway IDs.

**Figure 10:**
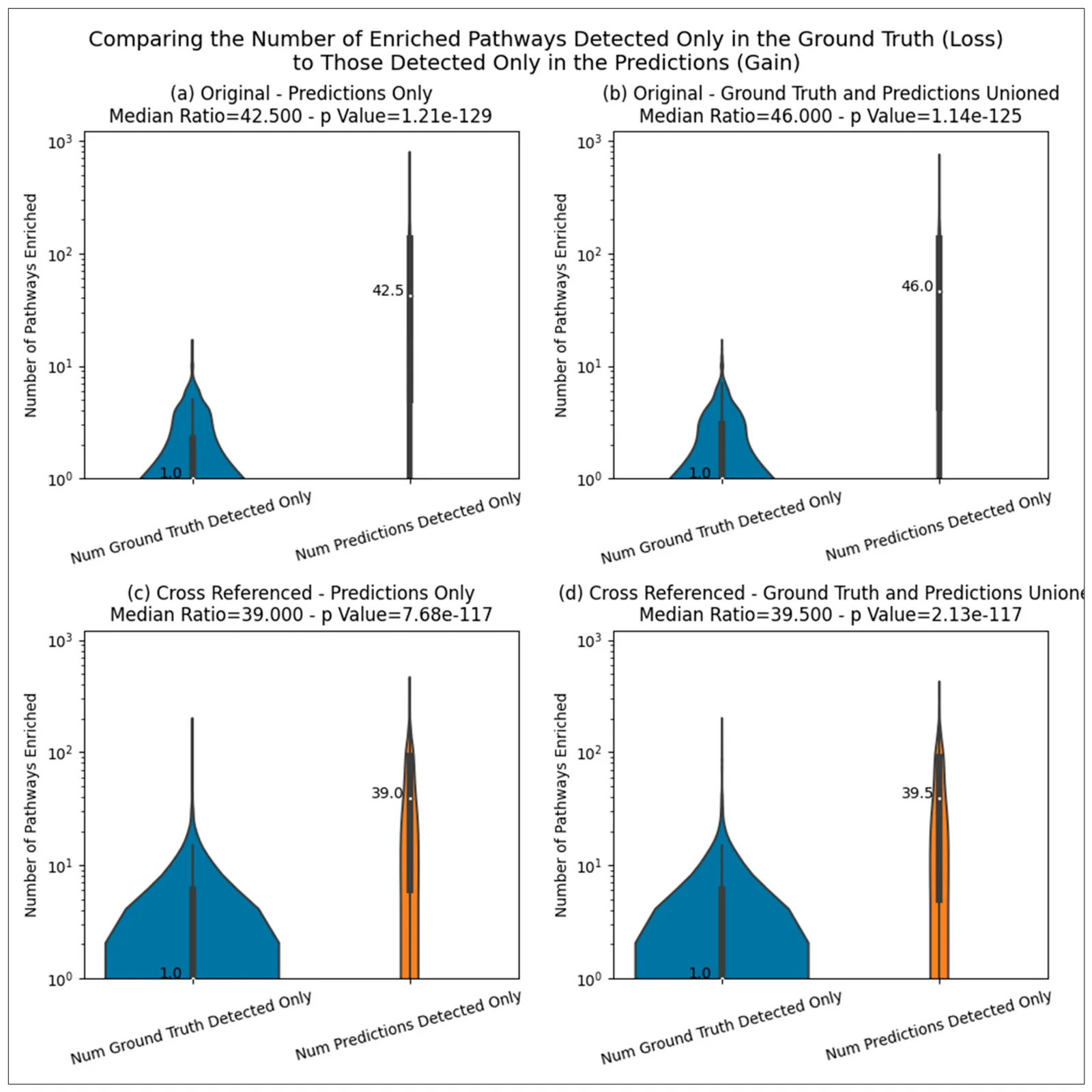
Comparing the distribution of information loss to that of information gain across MW datasets with more than 0 pathways enriched for both ground truth and predictions. We see that MW datasets experience far more information gain than information loss on average. Results are for unique pathway definitions. The annotation types are defined as follows: Ground Truth Only: Known pathway annotations as defined in knowledge bases; Predictions Only: Predicted pathway annotations alone; Ground Truth and Predictions Unioned: The unioned set of ground truth pathway annotations and predicted pathway annotations. The analysis types are defined as follows: Original: Derived from MW datasets containing the knowledge base compound IDs as provided by the original metadata; Cross-referenced: Derived from MW datasets containing the larger set of knowledge base compound IDs as a result of chained cross-referencing. The number of analyses in the groups being compared is as follows: (**a**) 668 (**b**) 658 (**c**) 771 (**d**) 770.

While Table 2 shows the sums of information loss and information gain across MW datasets, Figure 10 compares the distribution of these values generated for each MW dataset. We observe a median value of 1 for the information loss for all combinations of analysis type and annotation type. Every combination also has a median information gain of about 40-fold compared to information loss, and the differences are all highly statistically significant. Figure S26 shows these results for individual pathway IDs. Figure S27 represents the difference in information gain and information loss as an upset plot. Figure S28 shows the same results as Figure S27 but for individual pathway IDs. The *p*-values in Figure 10 are from a Mann–Whitney U test. The *p*-values from the Wilcoxon signed rank test are as follows: (a) 1.72 × 10^−96^ (b) 1.37 × 10^−94^ (c) 1.62 × 10^−103^ (d) 4.63 × 10^−103^.

**Figure 11:**
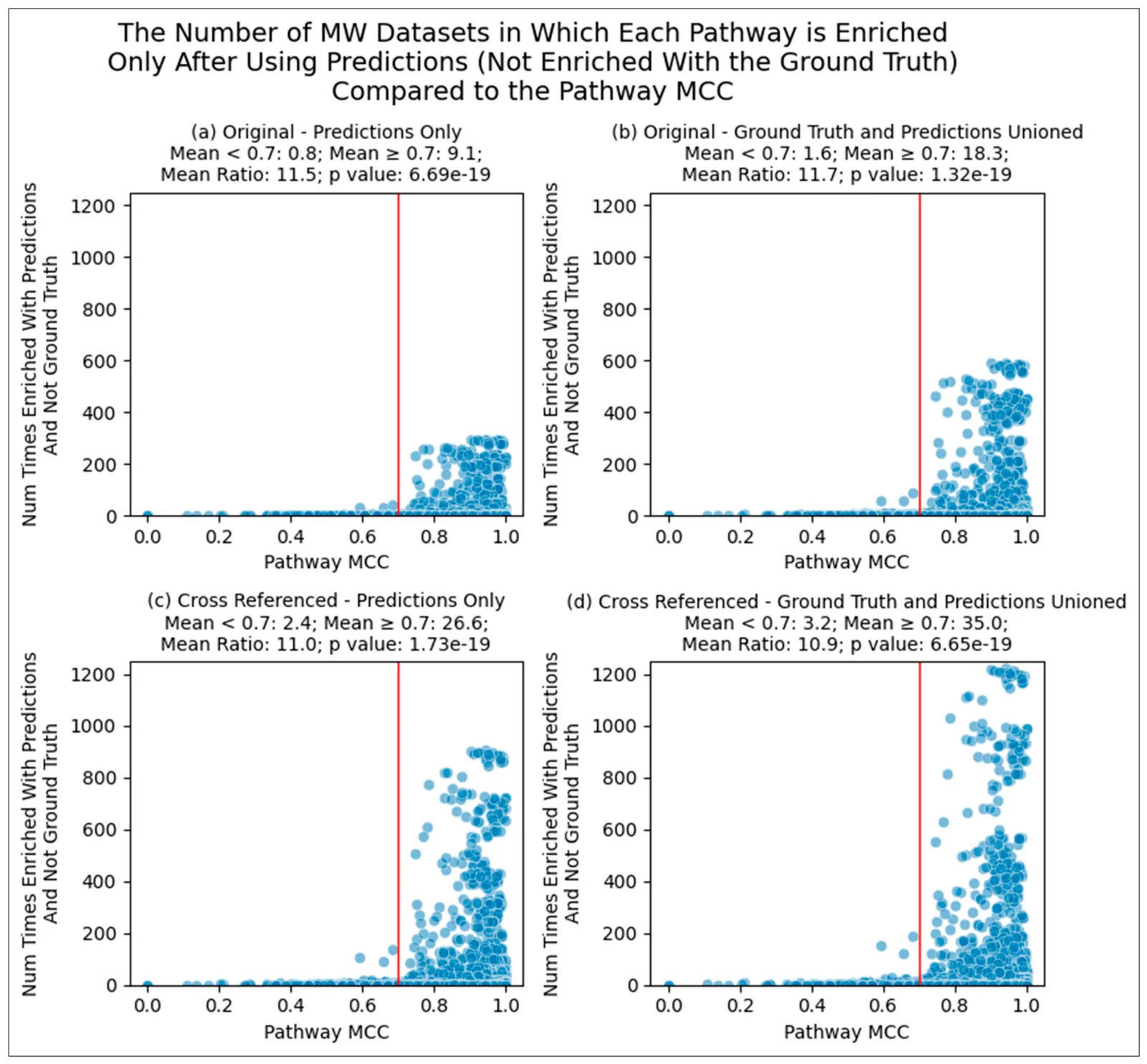
Comparing the pathway MCC to the number of MW datasets wherein each pathway was not enriched when using ground truth annotations but was enriched when using predicted annotations. The red line is drawn at overall MCC = 0.7. Each point is a different pathway enriched in at least one dataset. We see that the potential for a pathway to be enriched increases substantially when its MCC is at least 0.7. Results are for unique pathway definitions. The annotation types are defined as follows: Ground Truth Only: Known pathway annotations as defined in knowledge bases; predictions only: Predicted pathway annotations alone; Ground Truth and Predictions Unioned: The unioned set of ground truth pathway annotations and predicted pathway annotations. The analysis types are defined as follows: Original: Derived from MW datasets containing the knowledge base compound IDs as provided by the original metadata; Cross-referenced: Derived from MW datasets containing the larger set of knowledge base compound IDs as a result of chained cross-referencing. The numbers of pathways in each group compared are as follows: (**a**) 380 below, 5669 above; (**b**) 380 below, 5669 above; (**c**) 387 below, 5703 above; (**d**) 388 below, 5703 above.

In Figure 11, each pathway’s overall MCC, as obtained in the work of Huckvale and Moseley [38], is plotted against the sum of the number of times the pathway was enriched across all MW datasets only after adding predicted pathway annotations, but not enriched when using ground truth annotations alone. We see that for all combinations of original or cross-referenced datasets and predictions only or Predictions Unioned with Ground Truth, the number of times that pathways are enriched greatly increases when the predictive performance of pathways reaches an MCC of 0.7. We see an increase in the number of times enriched between pathways below an overall MCC of 0.7 and pathways equal to or above an overall MCC of 0.7, and the difference between the two groups is highly statistically significant (all *p*-values from a Mann–Whitney U test ≤ 10^−19^), with mean ratios ranging from 5.5- to 5.8-fold. We use mean ratios here since the median number of times enriched for pathways below an overall MCC of 0.7 was consistently 0, making it impossible to calculate ratios using the medians. These results illustrate a strong dependence of pathway enrichment detection on pathway prediction performance. Specifically, high enrichment does not occur until pathway prediction overall MCC is roughly 0.7 or above. While pathway MCC does have a weak relationship with pathway size [35,36,37], this does not change the impact of pathway MCC on pathway enrichment detection.

Table 3 demonstrates the coverage of metabolism in terms of the number of unique metabolites present within the metabolite-pathway annotations across MW datasets before and after using predictions. We see that the increase in the number of unique metabolites is greater for the original datasets compared to the cross-referenced datasets. Note that the number of datasets used to compute the summary statistics is less than 990 since datasets that had 0 metabolites present for both ground truth and predictions were filtered out. Table S3 shows these stats for all MW datasets unfiltered. Also note that it does not matter whether pathway IDs or pathway definitions are used since these counts are the union of pathway definitions (associated metabolites) and redundant pathway definitions leave the union unchanged.

Table 4 demonstrates information gain by quantifying the uniqueness of enriched pathways across MW datasets by calculating Jaccard indices of their associated definitions (associated metabolites). Specifically, this is calculated by summing the pathway uniqueness scores across enriched pathways in an MW dataset, the uniqueness score being calculated by averaging 1 minus the Jaccard index between its definition and the definition of every other enriched pathway in the dataset. Again, datasets with scores of 0 for both ground truth and predictions are filtered out (Table S4 shows these results unfiltered), and it does not matter whether pathway IDs or pathway definitions are used since redundant definitions would add a (1 − Jaccard index) = 0 to the average calculated for the uniqueness score.

#### 3.2.3. Comparison to Random Predictions

Table 5 shows the results of the CV analysis of the random prediction model. We see that the mean MCC of 0.1310 is far below the ML model’s MCC of 0.9036, demonstrating that use of machine learning far outperforms predicting pathways randomly. Figure S30 shows the distribution of the random prediction’s MCC across the 100 CV iterations.

**Figure 12:**
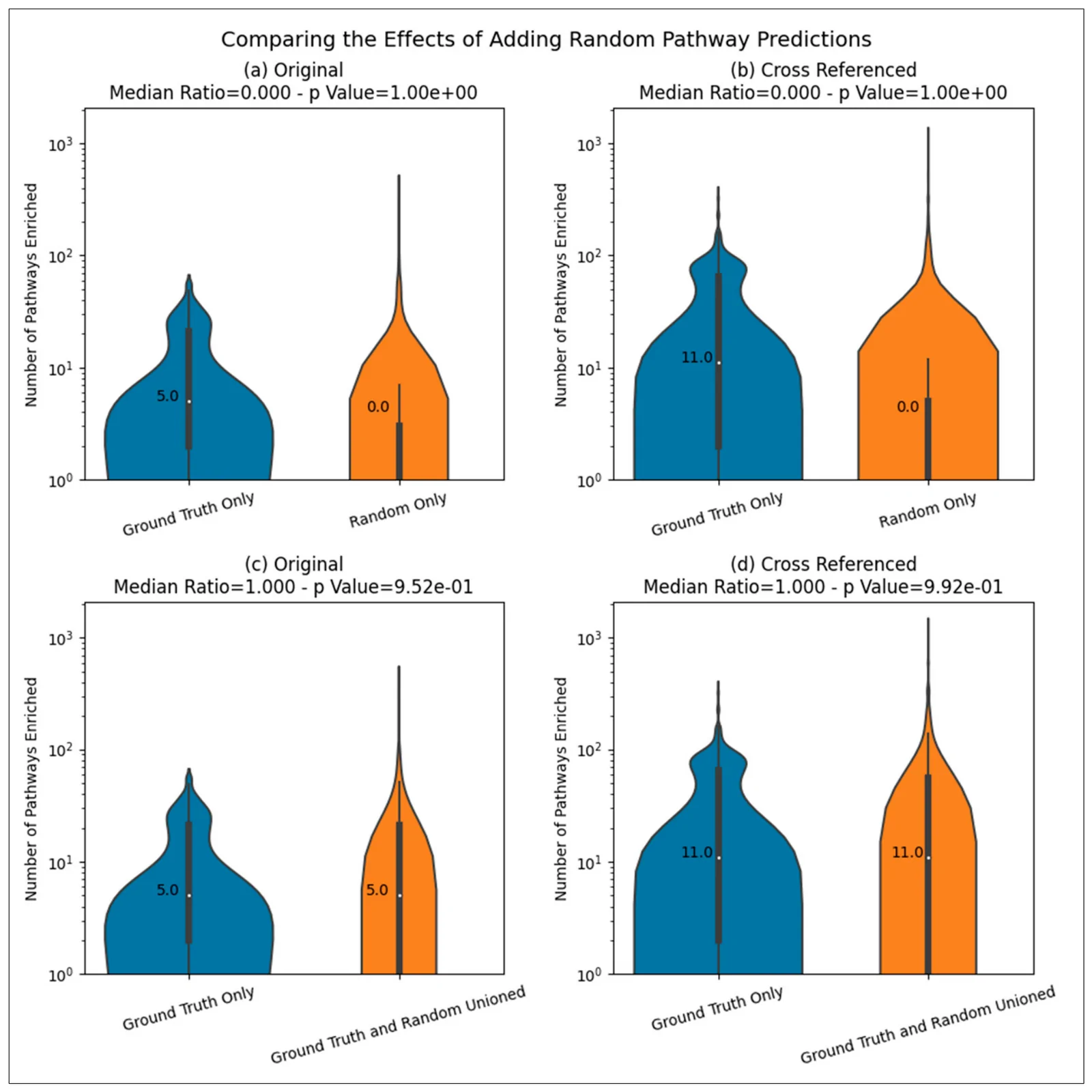
Comparing the number of pathways enriched for ground truth to that for random pathway predictions. We see that pathway predictions do not enhance PEA when predicted randomly. Results are for unique pathway definitions. The annotation types are defined as follows: Ground Truth Only: Known pathway annotations as defined in knowledge bases; Random Only: Randomly predicted pathway annotations alone; Ground Truth and Random Unioned: The unioned set of ground truth pathway annotations and randomly predicted pathway annotations. The analysis types are defined as follows: Original: Derived from MW datasets containing the knowledge base compound IDs as provided by the original metadata; Cross-referenced: Derived from MW datasets containing the larger set of knowledge base compound IDs as a result of chained cross-referencing.

Figure 12 compares the distribution of the number of pathways enriched across MW datasets when using ground truth metabolite-pathway annotations to that when using random metabolite-pathway predictions. When compared to random predictions only, we see that the median number of pathways enriched drops to 0 whether using the original or cross-referenced datasets. When unioning the random predictions with ground truth, we see there is no significant change from using the ground truth alone. These results show that not only does the random prediction model fail to accurately predict pathways, but its predictions subsequently fail to increase the number of detected pathways in metabolomics analyses. Analyses with 0 enriched pathways for both ground truth and predictions are filtered from the groups being compared. The sizes of the groups are as follows: (a) 593, (b) 639, (c) 593, (d) 639.

## 4. Discussion

Our results demonstrate the high utility of using machine learning to predict pathway annotations that enhance the results from PEA. Using an ML model with the highest prediction performance to date, along with the highest number of pathways capable of being predicted, we greatly increased the number of pathway annotations and pathways that are detectable (Figure 4 and Figure 5). The quality of the model’s pathway predictions, whether predicting unique pathway definitions or pathway IDs, is indicated by its ability to increase the number of pathways detected, i.e., pathways being associated with changes in metabolites across test groups at a statistically significant extent (Figure 8). We expect the 8-fold increase in the number of enriched unique pathways to have high potential for expanding the biological and biomedical interpretability and insight of metabolomics datasets.

We consider the ability to accurately predict a higher number of pathway annotations to be a substantial milestone in resolving both the lack of compound IDs in metabolomics datasets and the lack of pathway annotations for the corresponding metabolites in knowledge bases such as KEGG, MetaCyc, and Reactome. We demonstrate that the issues surrounding the lack of compound IDs and associated pathway IDs can be ameliorated by the combination of using our metabolite-pathway prediction model and chaining cross-references. However, we further demonstrate increases in the number of enriched pathways far beyond what cross-referencing can accomplish alone (Figure 6). From Figure 8, we see an over 7-fold increase in enriched pathways whether or not cross-referencing is performed in the pipeline. We also see an over 7-fold increase in enriched pathways whether or not ground truth annotations are available at all. Our metabolite-pathway prediction model is especially useful for researchers who either do not have access to compound ID cross-references or do not have access to pathway annotations at all. Our model is also very useful for duplicate metabolite entries of different compound IDs where one ID has pathway annotations, and the other does not, or where intra-knowledge base cross-references are not provided by the knowledge base. These findings are additionally useful when a compound ID is not available since our model can predict pathway annotations from the chemical structure information alone. This utility is emphasized in Figure 7, which shows that both predictions with ground truth and predictions alone detect pathways comparably well.

While the ML model’s predictions improve the results of PEA overall, there are some cases of information loss alongside the information gain (Figure 9 and Table 2). Given that the amount of information gain is about 40-fold higher than information loss (Figure 10), we expect the few cases of information loss to be attributed mainly to inaccuracies in the predictions of the model. This is somewhat corroborated by the higher gains in Reactome vs. KEGG and MetaCyc, with Reactome pathway predictions having an overall MCC of 0.94 versus 0.87 for KEGG and 0.88 for MetaCyc [35,36,37,38], providing evidence that more accurate predictions result in more enriched pathways. More specifically, Figure 11 shows that a pathway’s potential to be enriched increases between 10.9 and 11.5 fold when reaching a predictive performance of at least 0.7 overall MCC. In addition, we have compared our model’s performance in predicting metabolite-pathway annotations to predicting pathways based on random chance (Table 5). We demonstrated that our extreme classification model far outperforms the random prediction model both in MCC and its subsequent ability to enhance PEA (Figure 12). This is consistent with our results in Figure 11 showing that higher MCC pathways are detected more frequently in metabolic analyses than lower MCC pathways.

As of now, we recommend that researchers analyzing metabolomics datasets try both the ground truth annotations with chaining cross-references, if they have access to them, as well as our model’s predictions to gain the largest number of enriched pathways while avoiding any possible information loss. We anticipate that further improvements to the performance and generalizability of the model, especially to increase the number of pathways with an overall MCC ≥ 0.7, will likely result in higher information gain, further improving the interpretability of and insight from metabolomics analyses. Beyond information gain in the increased number of enriched pathways, we also see a gain in the amount of unique metabolic information (Table 4).

## 5. Conclusions

Our results demonstrate the utility of an ML model that predicts pathway annotations. The increased amount of pathway annotations greatly expands the potential discovery and interpretability of metabolomics datasets via PEA. However, the utility of PEA in biological interpretability depends on many extrinsic factors that affect data quality, including appropriateness of experimental design, presence of confounding factors, quality of the experiment implementation, and many others. All that is demonstrated here is an approach to improve PEA itself for metabolomics datasets.

## Figures and Tables

**Table 1:** Comparing the number of pathways available in different contexts.

#Known Pathway IDs	#Predictable Pathway IDs	#Known Unique Pathway Definitions	#Predictable Unique Pathway Definitions
23,110	22,265	8574	8517

**Table 2:** The amount of information gain and information loss summed across all enriched pathways and MW datasets with more than 0 pathways enriched for both ground truth and predictions. Results are for unique pathway definitions. The annotation types are defined as follows: Ground Truth Only: Known pathway annotations as defined in knowledge bases; Predictions Only: Predicted pathway annotations alone; Ground Truth and Predictions Unioned: The unioned set of ground truth pathway annotations and predicted pathway annotations. The analysis types are defined as follows: Original: Derived from MW datasets containing the knowledge base compound IDs as provided by the original metadata; Cross-referenced: Derived from MW datasets containing the larger set of knowledge base compound IDs as a result of chained cross-referencing.

Analysis Type	Annotation Type	Num Ground Truth Detected Only	Num Predictions Detected Only	Num Both Detected	Total Detected	Num Lost %	Num Gained %	Unchanged %	Num Analyses
Cross Referenced	Ground Truth and Predictions Unioned	3608	48,235	20,670	72,513	5.0	66.5	28.5	770
	Predictions Only	3572	48,394	20,706	72,672	4.9	66.6	28.5	771
Original	Ground Truth and Predictions Unioned	1033	52,063	65,14	59,610	1.7	87.3	10.9	658
	Predictions Only	1024	52,121	65,23	59,668	1.7	87.4	10.9	668

**Table 3:** The number of unique metabolites as part of metabolite-pathway annotations across MW datasets. Datasets with 0 metabolites present for both ground truth and predictions are filtered out. We see that incorporating predictions substantially increases the number of unique metabolites in MW datasets. The annotation types are defined as follows: Ground Truth Only: Known pathway annotations as defined in knowledge bases; Ground Truth and Predictions Unioned: The unioned set of ground truth pathway annotations and predicted pathway annotations. The analysis types are defined as follows: Original: Derived from MW datasets containing the knowledge base compound IDs as provided by the original metadata; Cross-referenced: Derived from MW datasets containing the larger set of knowledge base compound IDs as a result of chained cross-referencing.

Analysis Type	Annotation Type	Mean	Median	Max	Num Analyses
Original	Ground Truth	3784.7	4878.5	6501	502
Original	Predictions Unioned	10,062.0	11,179.0	18,411	502
Cross Referenced	Ground Truth	8862.0	9708.0	18,141	548
Cross Referenced	Predictions Unioned	9468.8	10,284.5	18,424	548

**Table 4:** Sum of 1 minus average Jaccard index between each enriched pathway definition and every other enriched pathway definition. Datasets with 0 enriched pathways in both ground truth and predictions are filtered out. We see that incorporating pathway predictions increases the uniqueness of pathways according to their associated metabolites. The annotation types are defined as follows: Ground Truth Only: Known pathway annotations as defined in knowledge bases; Ground Truth and Predictions Unioned: The unioned set of ground truth pathway annotations and predicted pathway annotations. The analysis types are defined as follows: Original: Derived from MW datasets containing the knowledge base compound IDs as provided by the original metadata; Cross-referenced: Derived from MW datasets containing the larger set of knowledge base compound IDs as a result of chained cross-referencing.

Analysis Type	Annotation Type	Mean	Median	Max	Num Analyses
Original	Ground Truth	13.3	8.4	64.3	502
Original	Predictions Unioned	125.0	89.3	2574.5	502
Cross Referenced	Ground Truth	42.8	24.0	530.9	548
Cross Referenced	Predictions Unioned	133.0	97.0	1002.9	548

**Table 5:** Cross-validation MCC results for the random prediction model. We see the mean MCC of the random predictor is far below that of the ML model.

Mean MCC	Median MCC	Standard Deviation
0.1310	0.1310	0.0019

## Data Availability

All data and code for reproducing the results of this manuscript are available in the following Figshare item: https://doi.org/10.6084/m9.figshare.30645875 (18 November 2025).

## Supplementary Materials

The following supporting information can be downloaded at https://www.mdpi.com/article/10.3390/metabo16080545/s1, Table S1: The number of MW datasets filtered by reason for filtering; Table S2: The amount of information gain and information loss summed across all enriched pathways and MW datasets. Results are for individual pathway IDs; Table S3: The number of unique metabolites as part of metabolite-pathway annotations across MW datasets; Table S4: Sum of 1 minus average Jaccard index between enriched pathway definitions and every other pathway definition; Table S5: Hyperparameters used for the ML model; Figure S1: Violin plots comparing the number of detectable pathways and total number of metabolite-pathway annotations for the combined data; Figure S2: The median across MW datasets of the number of pathways that are detectable (associated with at least 15 metabolites) and the total number of metabolite-pathway associations. Results are for individual pathway IDs; Figure S3: Comparing the median number of detectable pathways and total metabolite-pathway annotations between original and cross-referenced MW datasets and between ground truth and predictions for each knowledge base. Results are for unique pathway definitions; Figure S4: Same as Figure S3 but for individual pathway IDs; Figure S5: Per-dataset view of the amount of increase in the number of detectable pathways and the total number of metabolite-pathway associations between ground truth annotations and the ground truth unioned with the predictions. Note that the axes are on different scales across the 4 scatterplots. Each point is a separate MW dataset. Results are for individual pathway IDs; Figure S6: Per-analysis view of the change in the number of detectable pathways between the ground truth and that unioned with predictions for each knowledge base using cross-referenced MW datasets. Results are for unique pathway definitions; Figure S7: Per-analysis view of the change in the number of detectable pathways between the ground truth and that unioned with predictions for each knowledge base using the original MW datasets. MetaCyc and Reactome are not shown here since there were no metabolite IDs available for these in the original datasets. Results are for unique pathway definitions; Figure S8: Per-analysis view of the change in the number of metabolite-pathway annotations between the ground truth and that unioned with predictions for each knowledge base using cross-referenced MW datasets. Results are for unique pathway definitions; Figure S9: Per-analysis view of the change in the number of metabolite-pathway annotations between the ground truth and that unioned with predictions for each knowledge base using the original MW datasets. MetaCyc and Reactome are not shown here since there were no metabolite IDs available for these in the original datasets. Results are for unique pathway definitions; Figure S10: Same as Figure S6 but for individual pathway IDs; Figure S11: Same as Figure S7 but for individual pathway IDs; Figure S12: Same as Figure S8 but for individual pathway IDs; Figure S13: Same as Figure S9 but for individual pathway IDs; Figure S14: The effect on the number of enriched pathways of adding metabolite ID cross-references to the MW datasets for each type of annotations. Results are for individual pathway IDs; Figure S15: The effect on the number of enriched pathways when unioning the predictions with the ground truth. Results are for individual pathway IDs; Figure S16: The increase in the number of enriched pathways when adding predicted annotations. Results are for individual pathway IDs; Figure S17: Comparing the distribution across MW analyses of the number of enriched pathways between the ground truth pathway annotations and those unioned with the predicted annotations for the cross-referenced MW datasets for each knowledge base. Combined results included for comparison. Results are for unique pathway definitions; Figure S18: Comparing the distribution across MW analyses of the number of enriched pathways between the ground truth pathway annotations and those unioned with the predicted annotations for the original MW datasets for each knowledge base. Note that MetaCyc and Reactome had no ground truth annotations available since their metabolite IDs were not available in the original MW datasets. Combined results included for comparison. Results are for unique pathway definitions; Figure S19: Same as Figure S17 but for individual pathway IDs; Figure S20: Same as Figure S18 but for individual pathways; Figure S21: The per-dataset change in the number of enriched pathways between the ground truth and the ground truth unioned with the predictions. Each point is a separate MW dataset. Results are for individual pathway IDs; Figure S22: Per-analysis view of the change in the number of enriched pathways between the ground truth pathway annotations and those unioned with the predicted annotations for each knowledge base on the cross-referenced MW datasets. Results are for unique pathway definitions; Figure S23: Per-analysis view of the change in the number of enriched pathways between the ground truth pathway annotations and those unioned with the predicted annotations for each knowledge base on the original MW datasets. Note that MetaCyc and Reactome are not shown because these had no metabolite IDs available in the original datasets. Results are for unique pathway definitions; Figure S24: Same as Figure S22 but for individual pathway IDs; Figure S25: Same as Figure S23 but for individual pathway IDs; Figure S26: Comparing the distribution of information loss to that of information gain across MW datasets. Results are for individual pathway IDs; Figure S27: Represents the information loss and information gain of enriched pathways between ground truth and predictions by showing the overlap of detected pathways. Results are for unique pathway definitions; Figure S28: Same as Figure S27 but for individual pathway IDs; Figure S29: Comparing the pathway MCC to the number of MW datasets wherein each pathway was not enriched when using ground truth annotations but was enriched when using predicted annotations. The red line is drawn at overall MCC = 0.7. Each point is a different pathway enriched in at least one dataset. Results are for individual pathway IDs; Figure S30: Distribution of the MCC across 100 CV iterations, evaluating the performance of the random prediction model’s ability to predict metabolite-pathway annotations.

## Abbreviations

The following abbreviations are used in this manuscript:

PEA	Pathway Enrichment Analysis
AEA	Annotation Enrichment Analysis
KEGG	Kyoto Encyclopedia of Genes and Genomes
InChI	IUPAC International Chemical Identifier
ML	Machine Learning
MLP	Multi-layer Perceptron
MCC	Matthew’s Correlation Coefficient
MW	Metabolomics Workbench
PCA	Principal Component Analysis
PC	Principal Component
GSEA	Gene Set Enrichment Analysis
