# Supplementary material for: High-Quality Predicted Metabolite-Pathway Annotations Expand Pathway Coverage and Increase Enriched Pathway Detection Across Metabolomics Datasets
Source: Metabolites. 2026 Jul 31;16(8):545. doi: 10.3390/metabo16080545 (PMC13515532; doi:10.3390/metabo16080545)
Supplement: Supplementary file 1 [file metabolites-16-00545-s001.zip › metabolites-4419525-supplementary.pdf]

Supplementary Materials
for

High-Quality Predicted Metabolite-Pathway Annotations Expand Pathway Coverage and Increase Enriched Pathway Detection Across Metabolomics Datasets

Erik D. Huckvale [1], P. Travis Thompson [1,2], Robert M. Flight [1,2,3] and Hunter N. B. Moseley [1,2,3,4,*]

[1] Markey Cancer Center, University of Kentucky, Lexington, KY 40506, USA; edhu227@uky.edu (E.D.H.); ptth222@uky.edu (P.T.T.); robert.flight@uky.edu (R.M.F.)
[2] Superfund Research Center, University of Kentucky, Lexington, KY 40506, USA
[3] Department of Molecular and Cellular Biochemistry, University of Kentucky, Lexington, KY 40506, USA
[4] Institute for Biomedical Informatics, University of Kentucky, Lexington, KY 40506, USA
* Correspondence: hunter.moseley@uky.edu

Filtering Counts

Table S1 - The number of MW datasets filtered by reason for filtering.

**Reason to Filter**	**Number of Datasets Filtered**
Neither KEGG nor PubChem compound ID field	2,356
Insufficient number of metabolites	900
Metabolite intensities on the wrong scale	113
Software failure	8
Unreadable file	5

Pre-GSEA

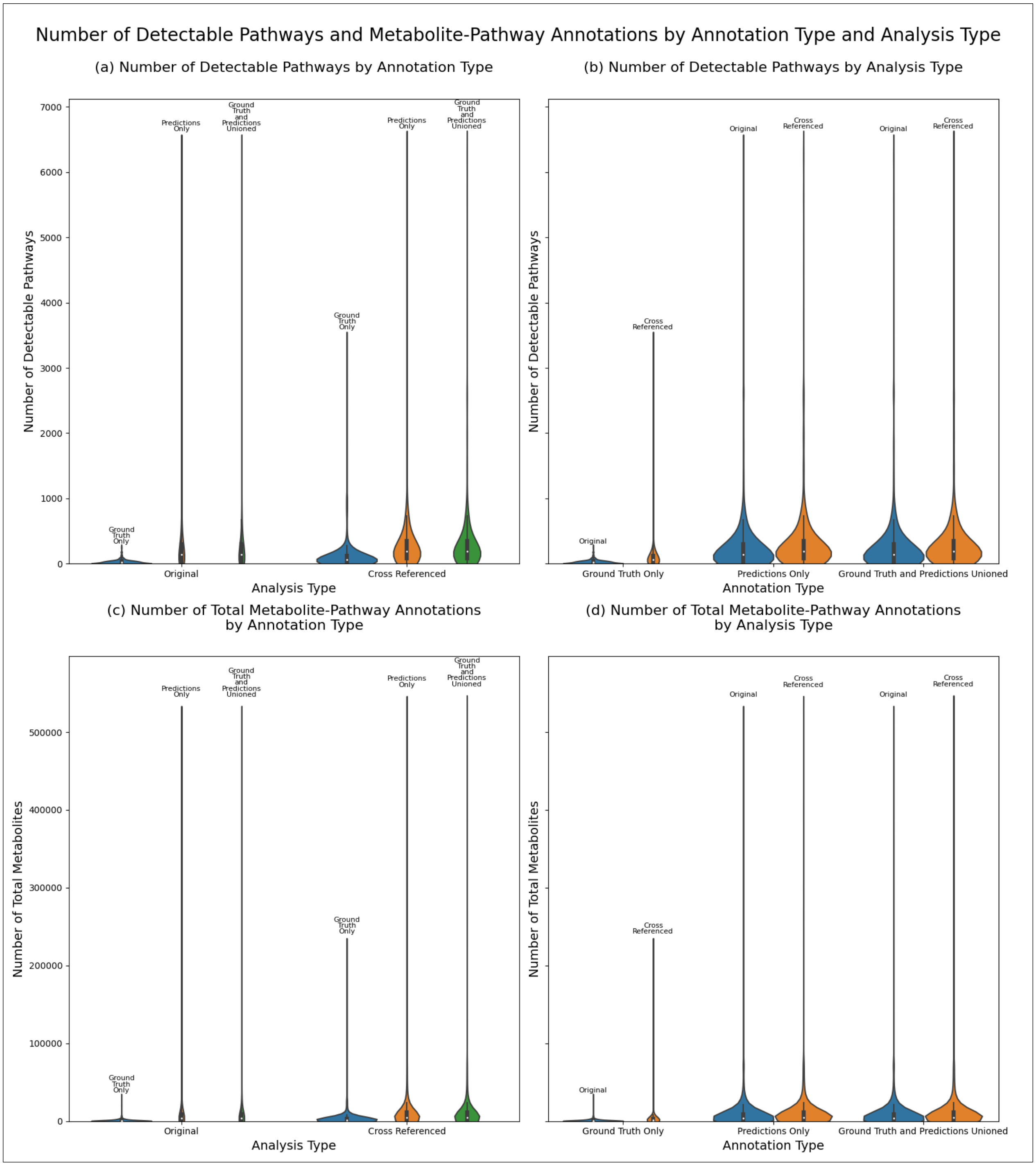

Figure S1 - Violin plots comparing the number of detectable pathways and total number of metabolite-pathway annotations for the combined data.

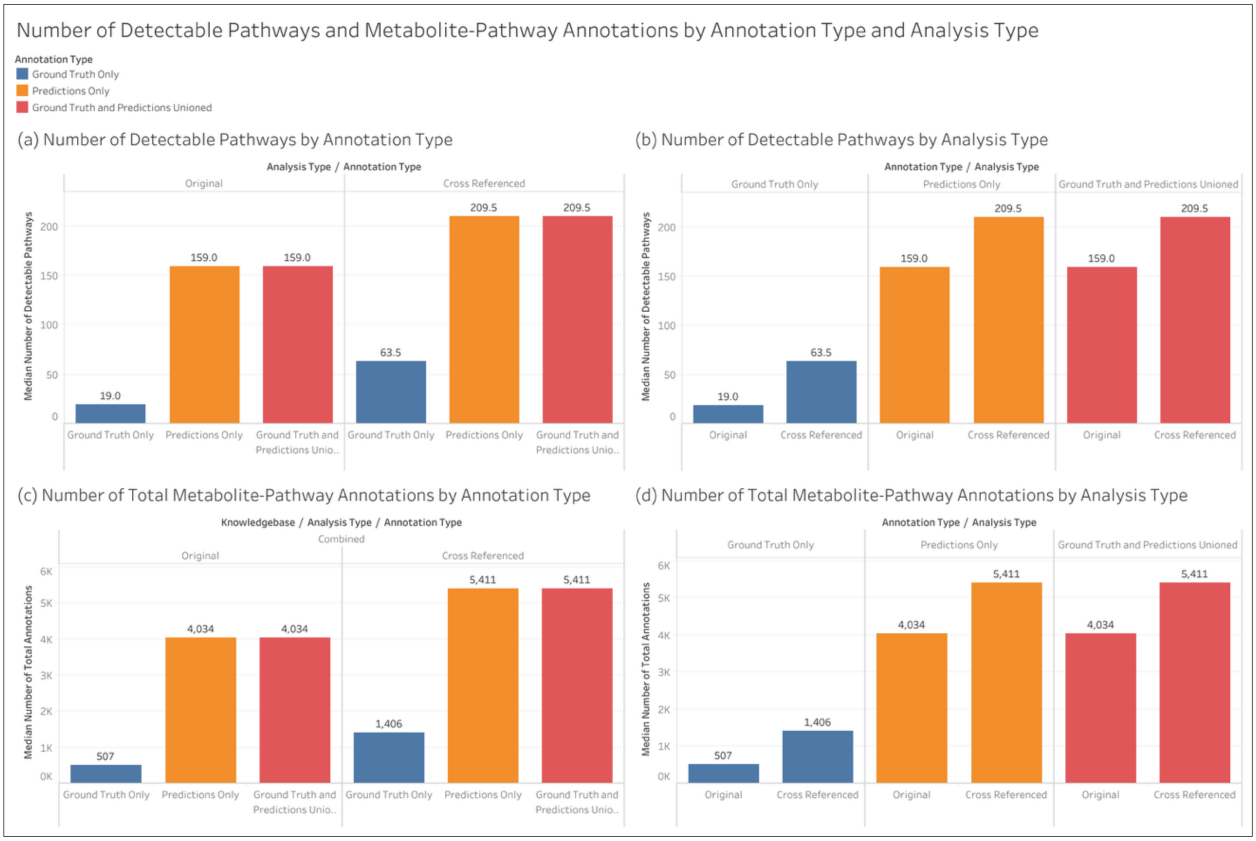

Figure S2 - The median across MW datasets of the number of pathways that are detectable (associated with at least 15 metabolites) and the total number of metabolite-pathway associations. Results are for individual pathway IDs.

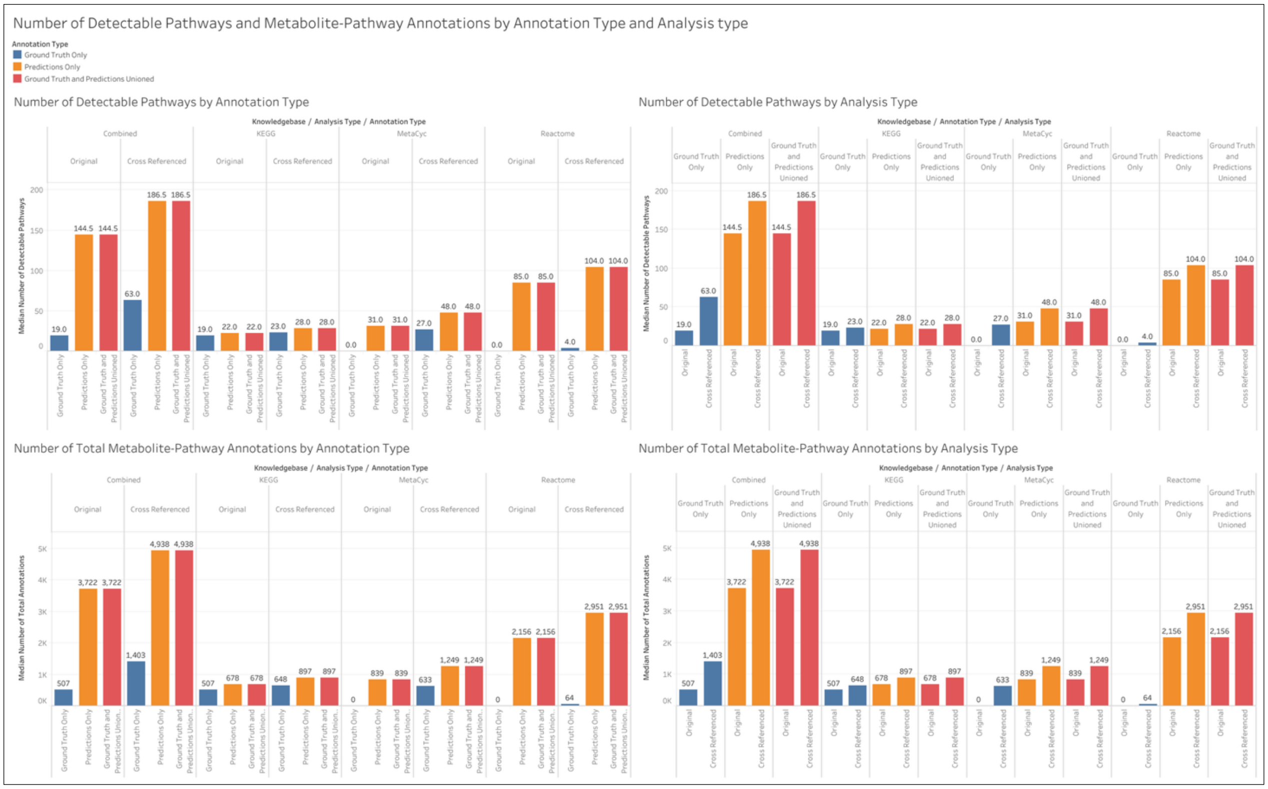

Figure S3 - Comparing the median number of detectable pathways and total metabolite-pathway annotations between original and cross-referenced MW datasets and between ground truth and predictions for each knowledgebase. Results are for unique pathway definitions.

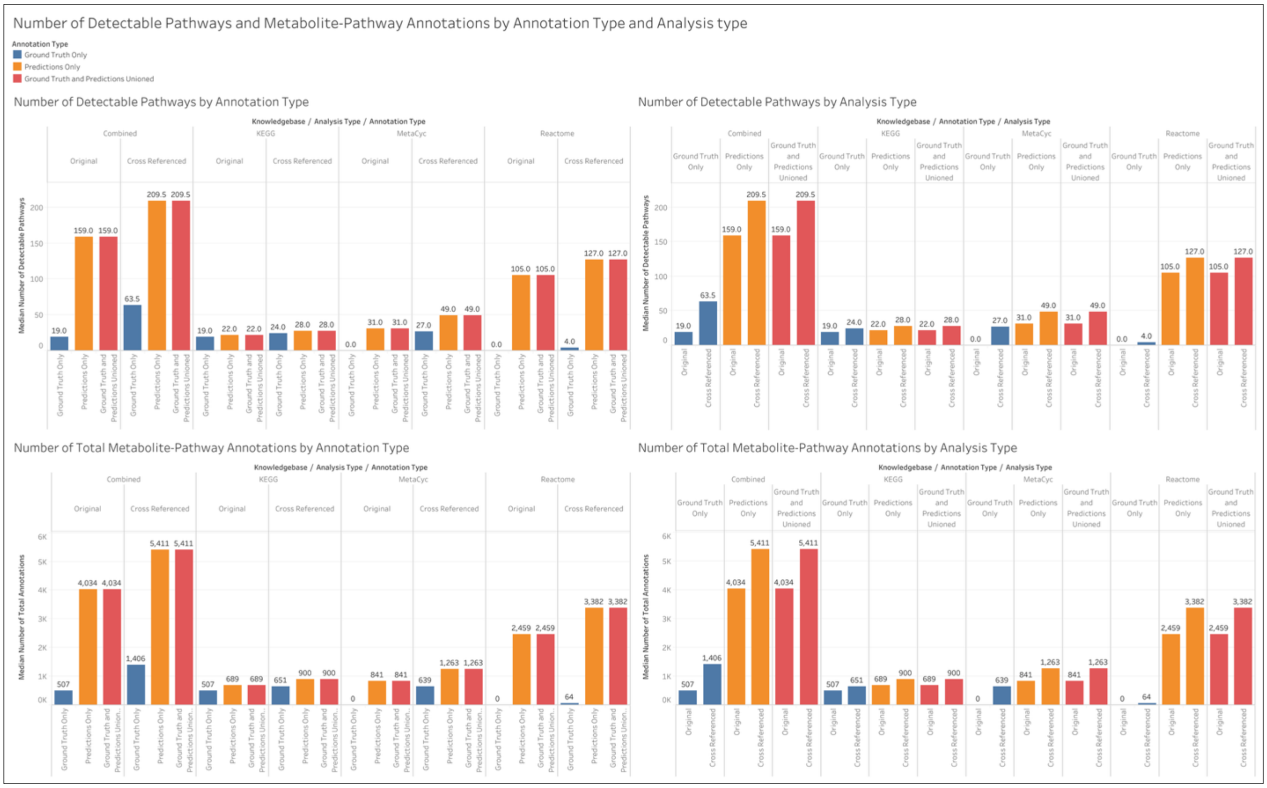

Figure S4 - Same as Figure S3 but for individual pathway IDs.

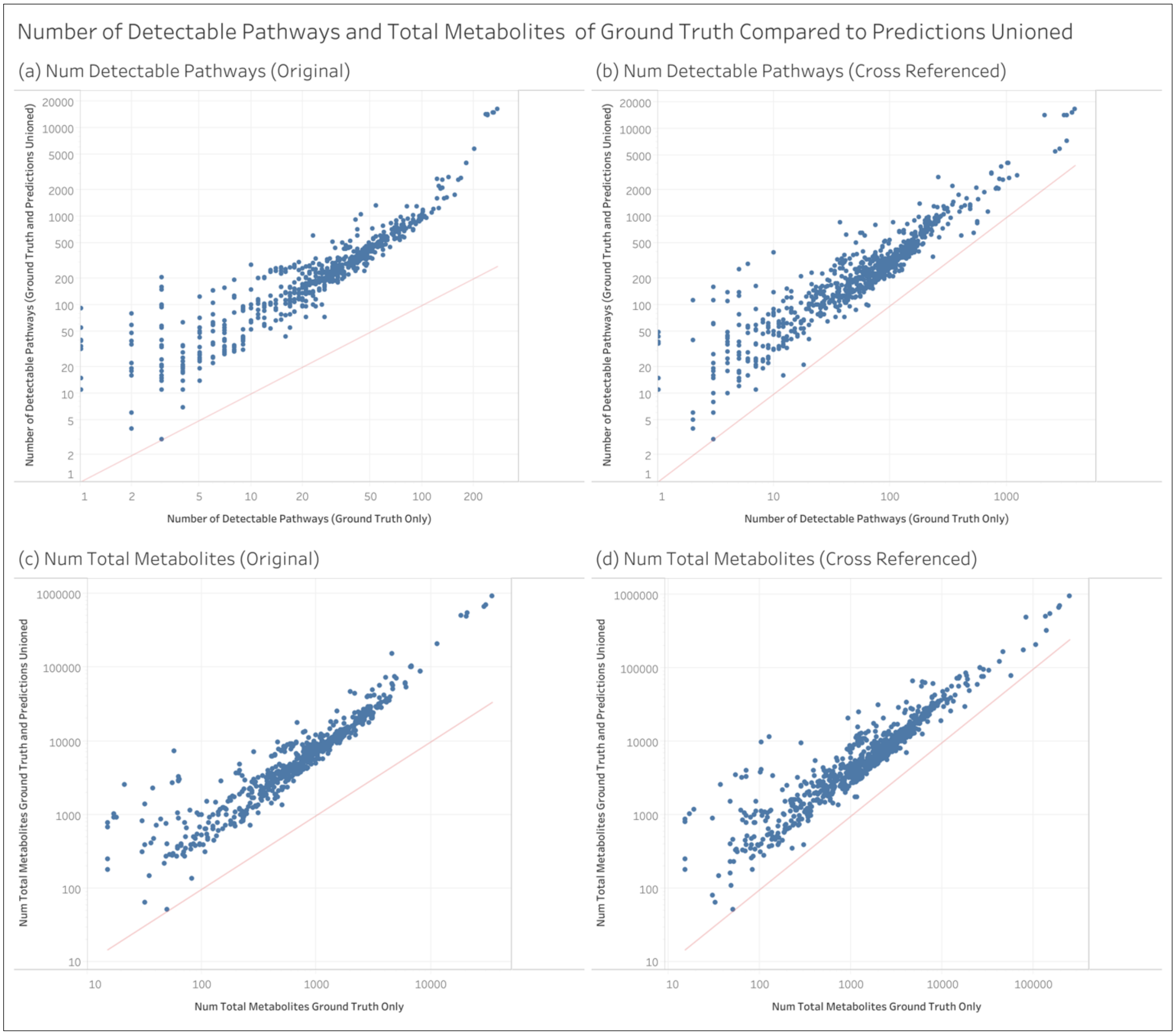

Figure S5 - Per dataset view of the amount of increase in the number of detectable pathways and the total number of metabolite-pathway associations between ground truth annotations and the ground truth unioned with the predictions. Note that the axes are on different scales across the 4 scatterplots. Each point is a separate MW dataset. Results are for individual pathway IDs.

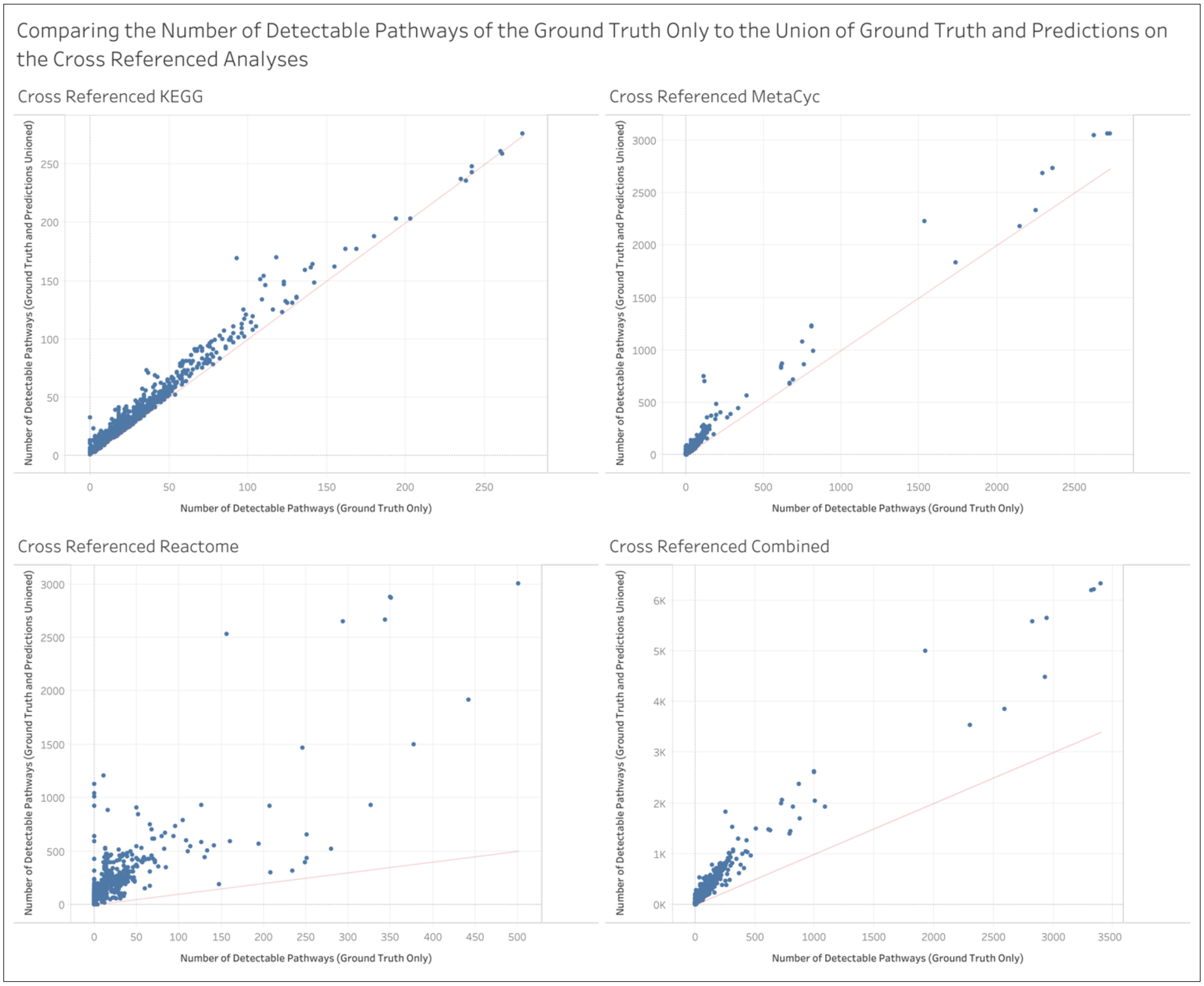

Figure S6 - Per analysis view of the change in the number of detectable pathways between the ground truth and that unioned with predictions for each knowledgebase using cross-referenced MW datasets. Results are for unique pathway definitions.

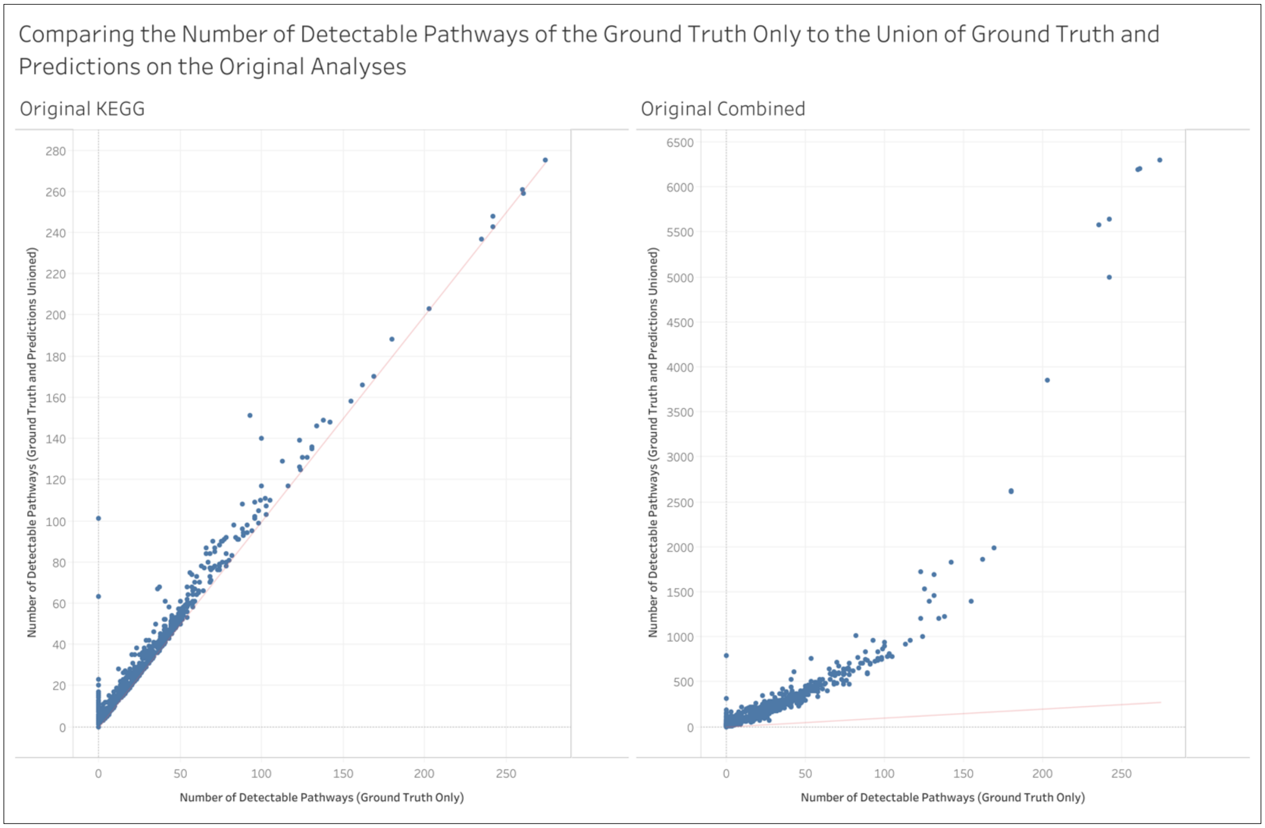

Figure S7- Per analysis view of the change in the number of detectable pathways between the ground truth and that unioned with predictions for each knowledgebase using the original MW datasets. MetaCyc and Reactome are not shown here since there were not any metabolite IDs available for these in the original datasets. Results are for unique pathway definitions.

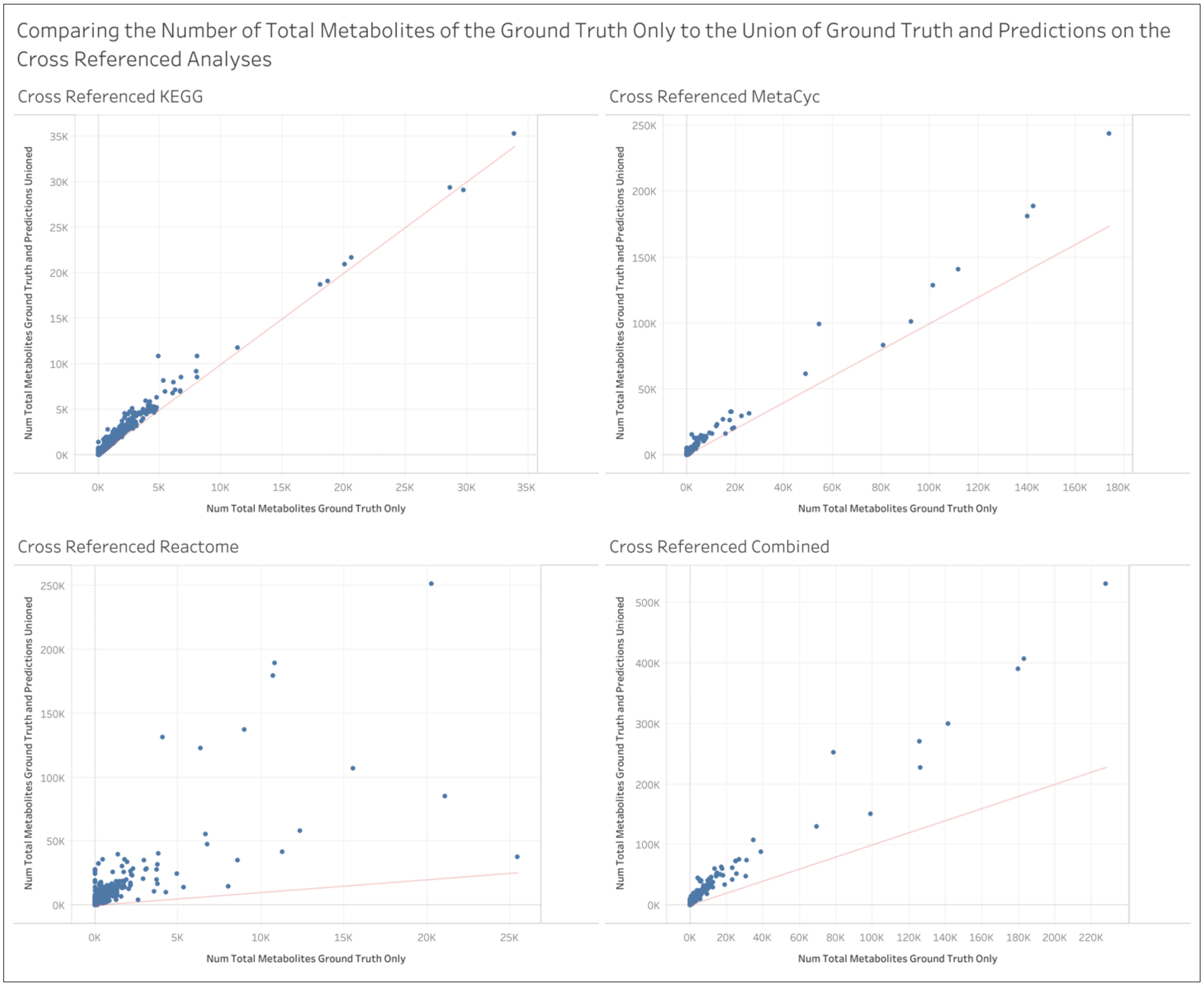

Figure S8- Per analysis view of the change in the number of metabolite-pathway annotations between the ground truth and that unioned with predictions for each knowledgebase using cross-referenced MW datasets. Results are for unique pathway definitions.

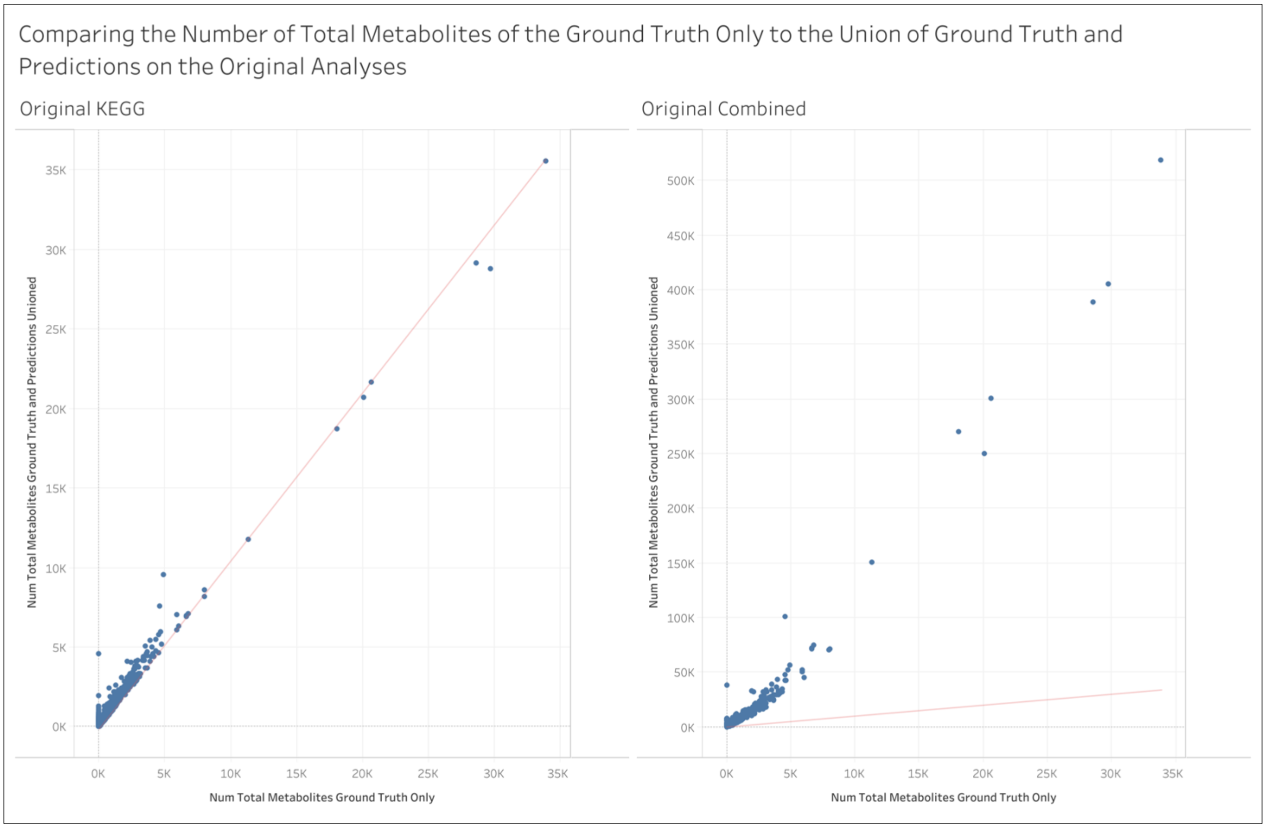

Figure S9- Per analysis view of the change in the number of metabolite-pathway annotations between the ground truth and that unioned with predictions for each knowledgebase using the original MW datasets. MetaCyc and Reactome are not shown here since there were not any metabolite IDs available for these in the original datasets. Results are for unique pathway definitions.

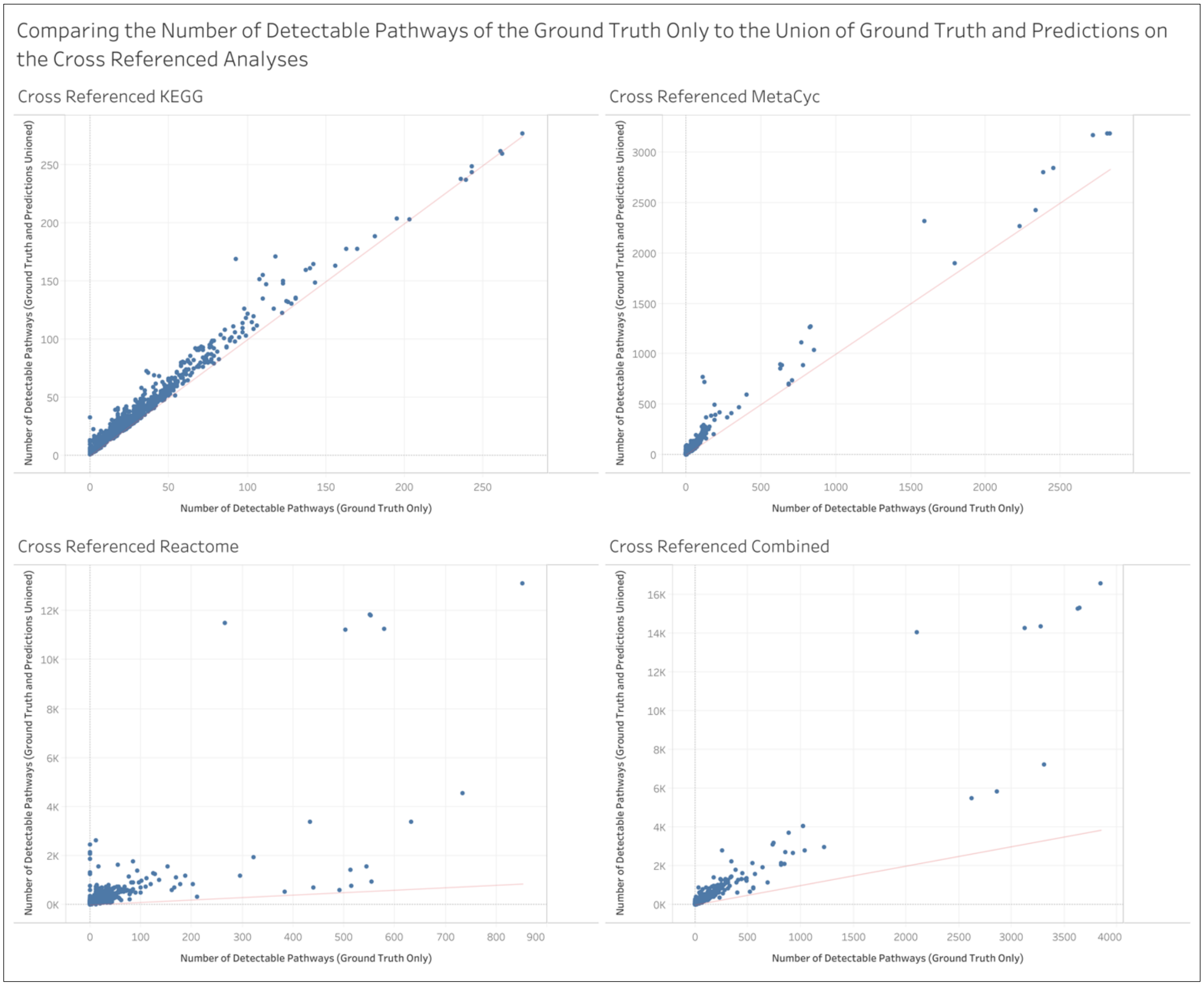

Figure S10 – Same as Figure S6 but for individual pathway IDs.

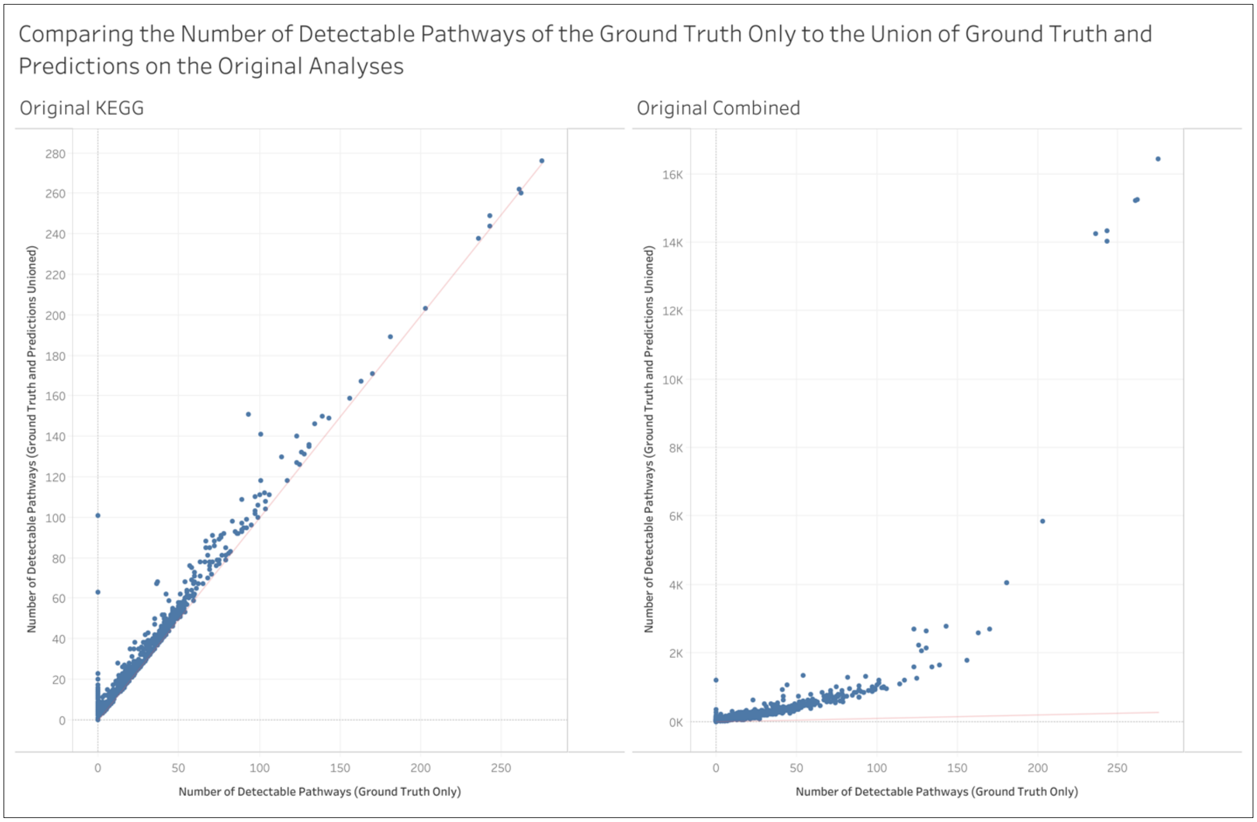

Figure S11 – Same as Figure S7 but for individual pathway IDs.

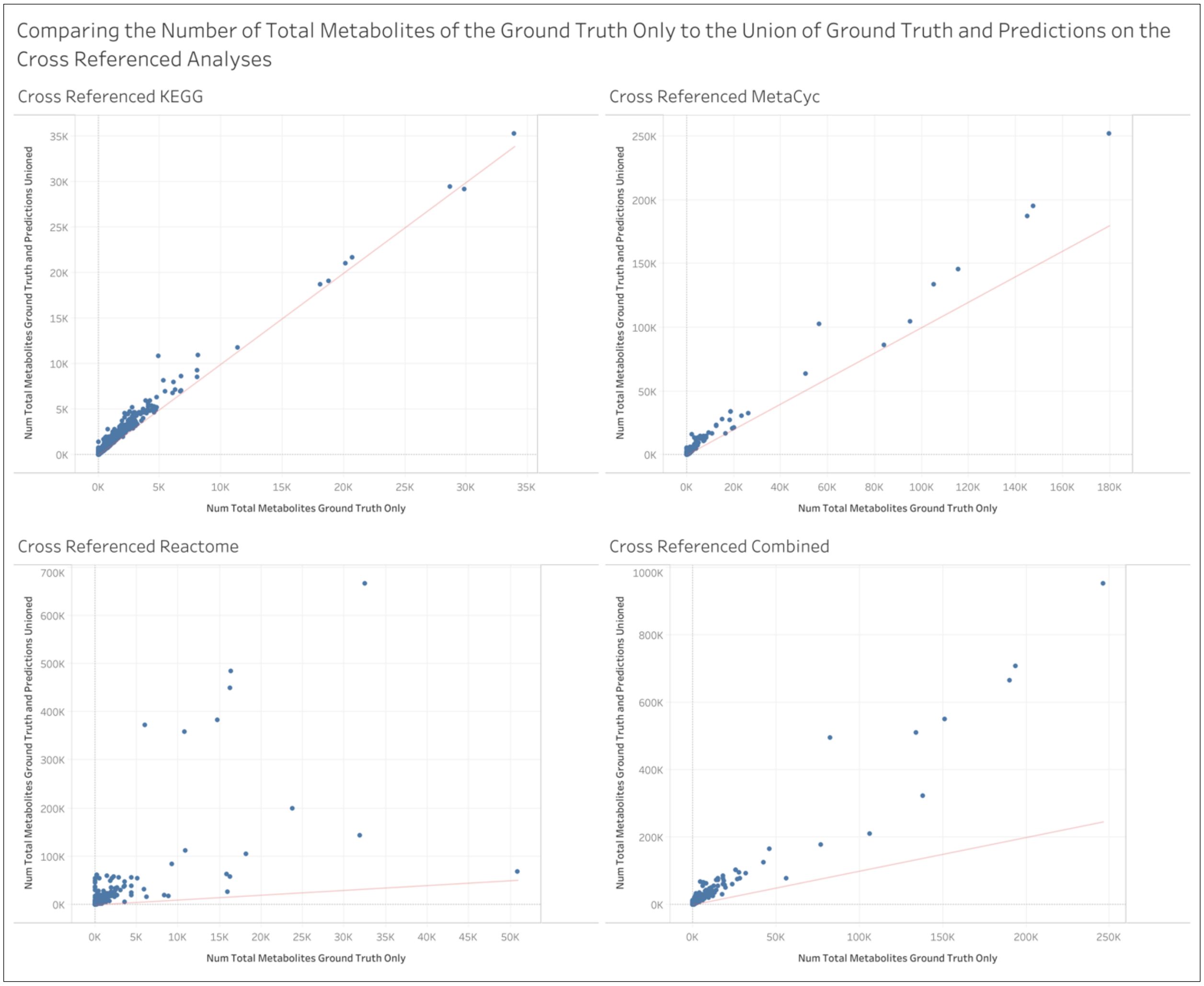

Figure S12 – Same as Figure S8 but for individual pathway IDs.

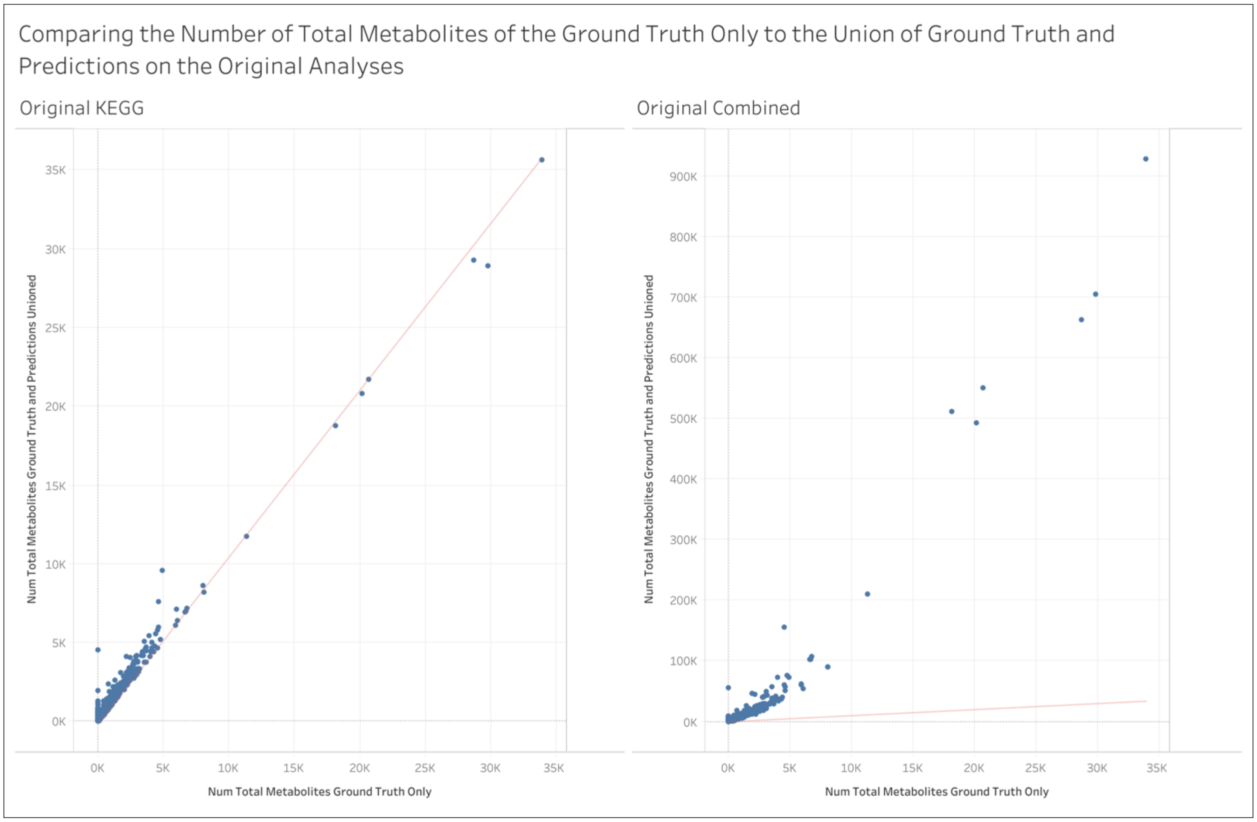

Figure S13 – Same as Figure S9 but for individual pathway IDs.

Post-GSEA

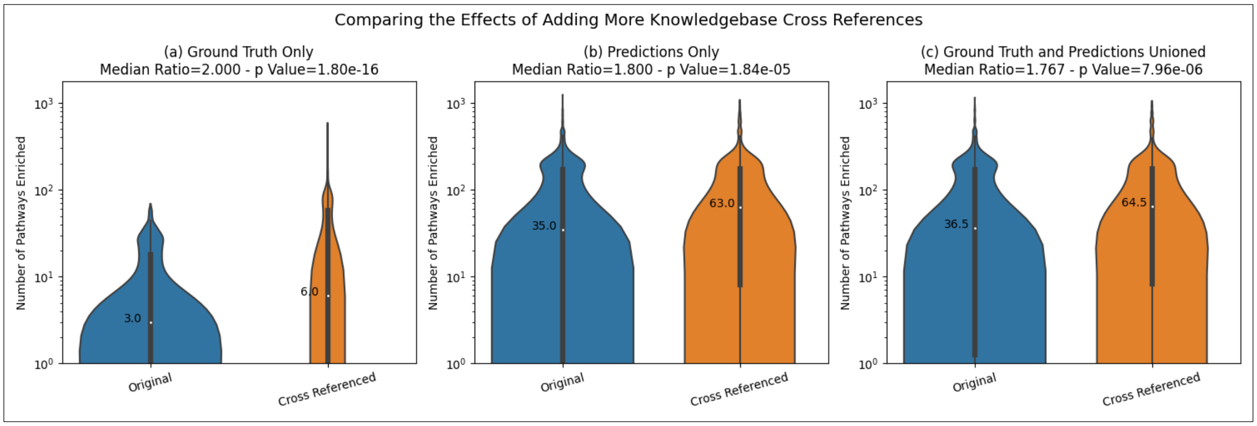

Figure S14 - The effect on the number of enriched pathways of adding metabolite ID cross references to the MW datasets for each type of annotations. Results are for individual pathway IDs.

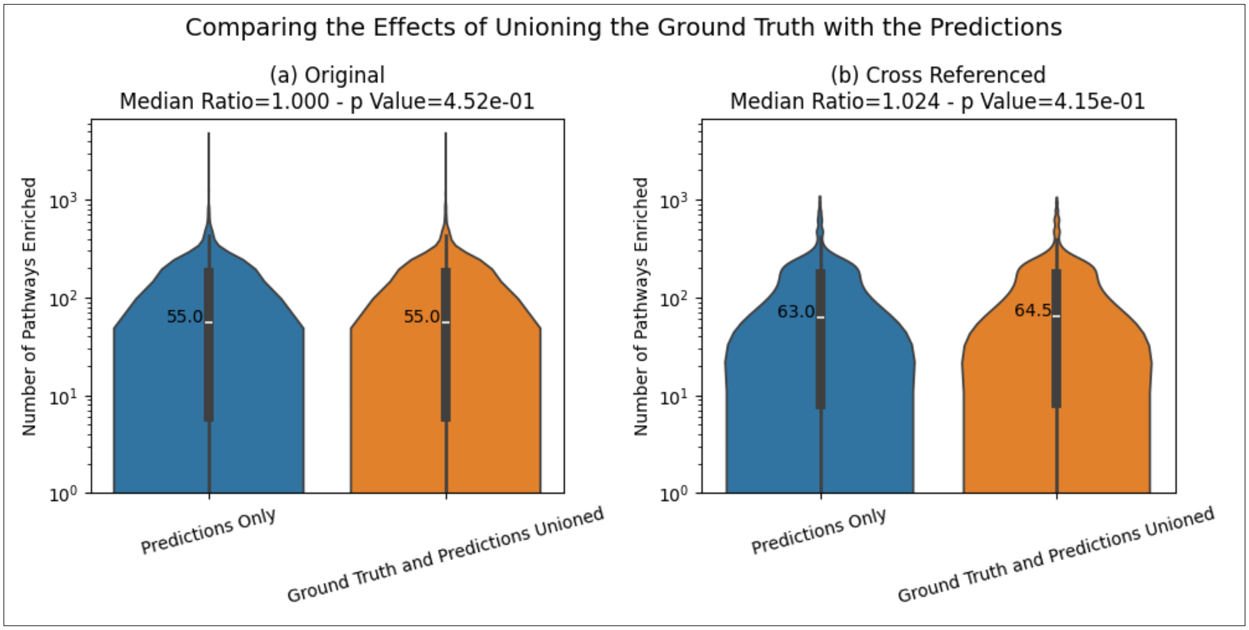

Figure S15 - The effect on the number of enriched pathways when unioning the predictions with the ground truth. Results are for individual pathway IDs.

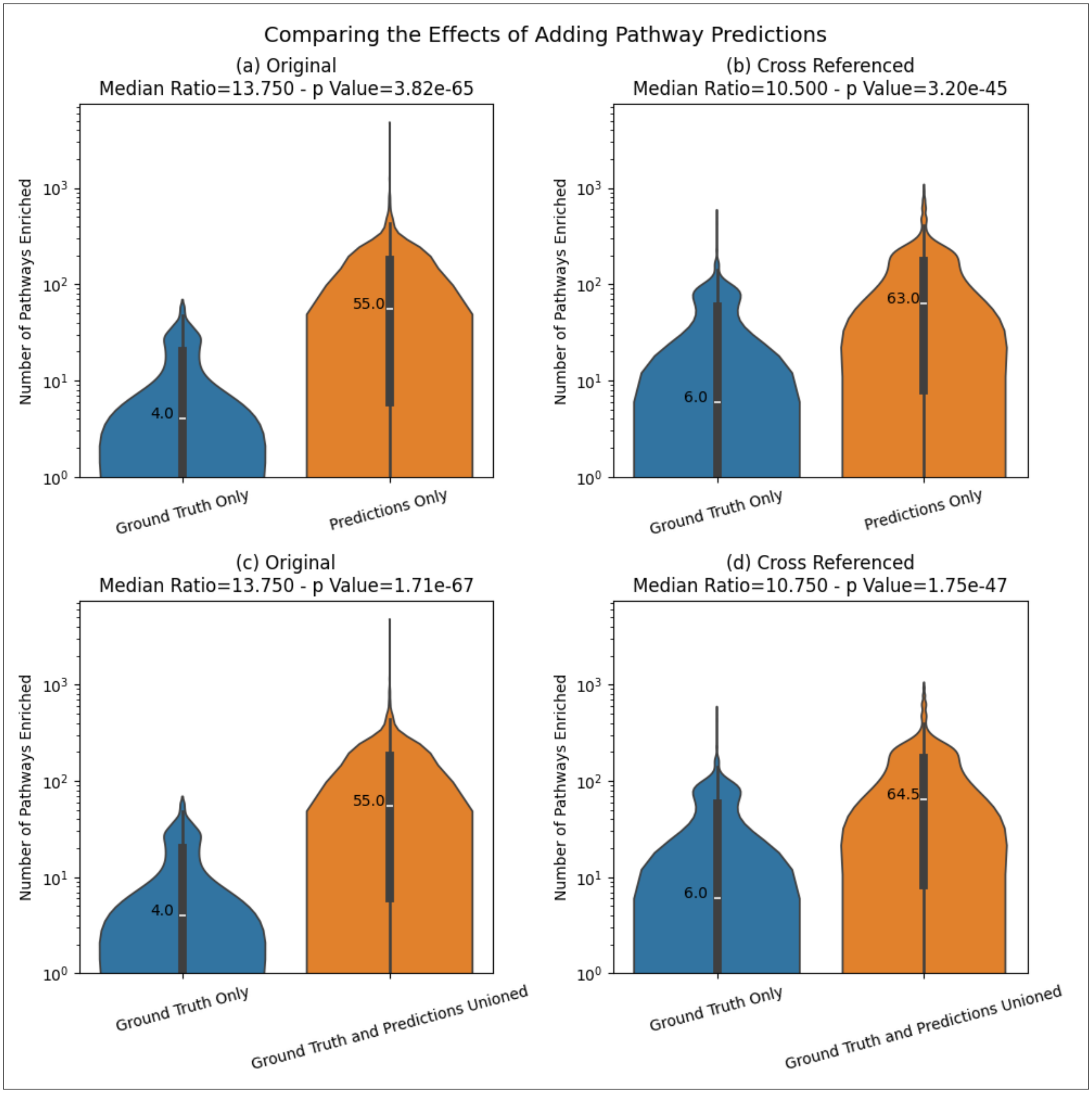

Figure S16 - The increase of the number of enriched pathways when adding predicted annotations. Results are for individual pathway IDs.

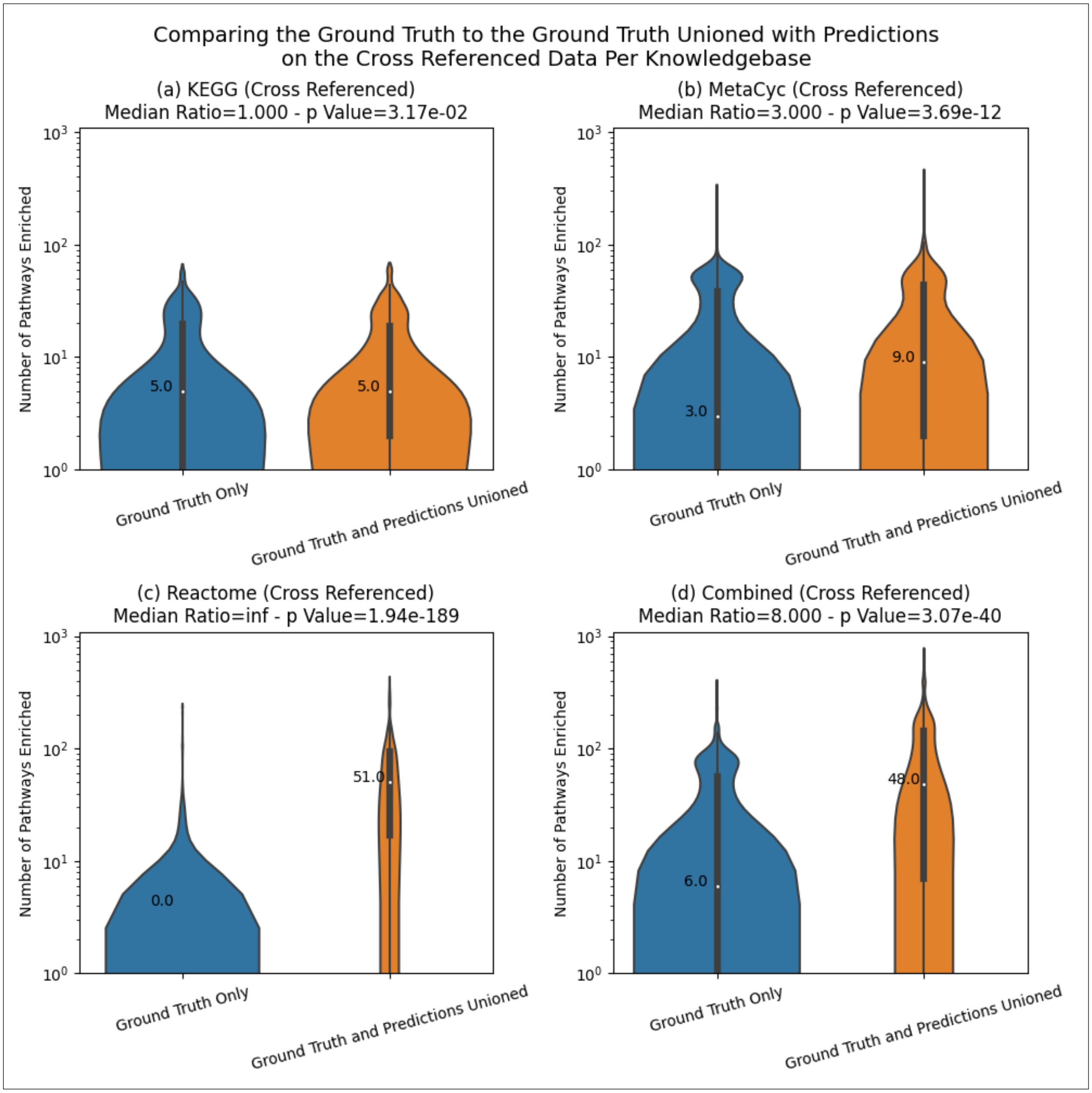

Figure S17 -Comparing the distribution across MW analyses of the number of enriched pathways between the ground truth pathway annotations and those unioned with the predicted annotations for the cross referenced MW datasets for each knowledgebase. Combined results included for comparison. Results are for unique pathway definitions.

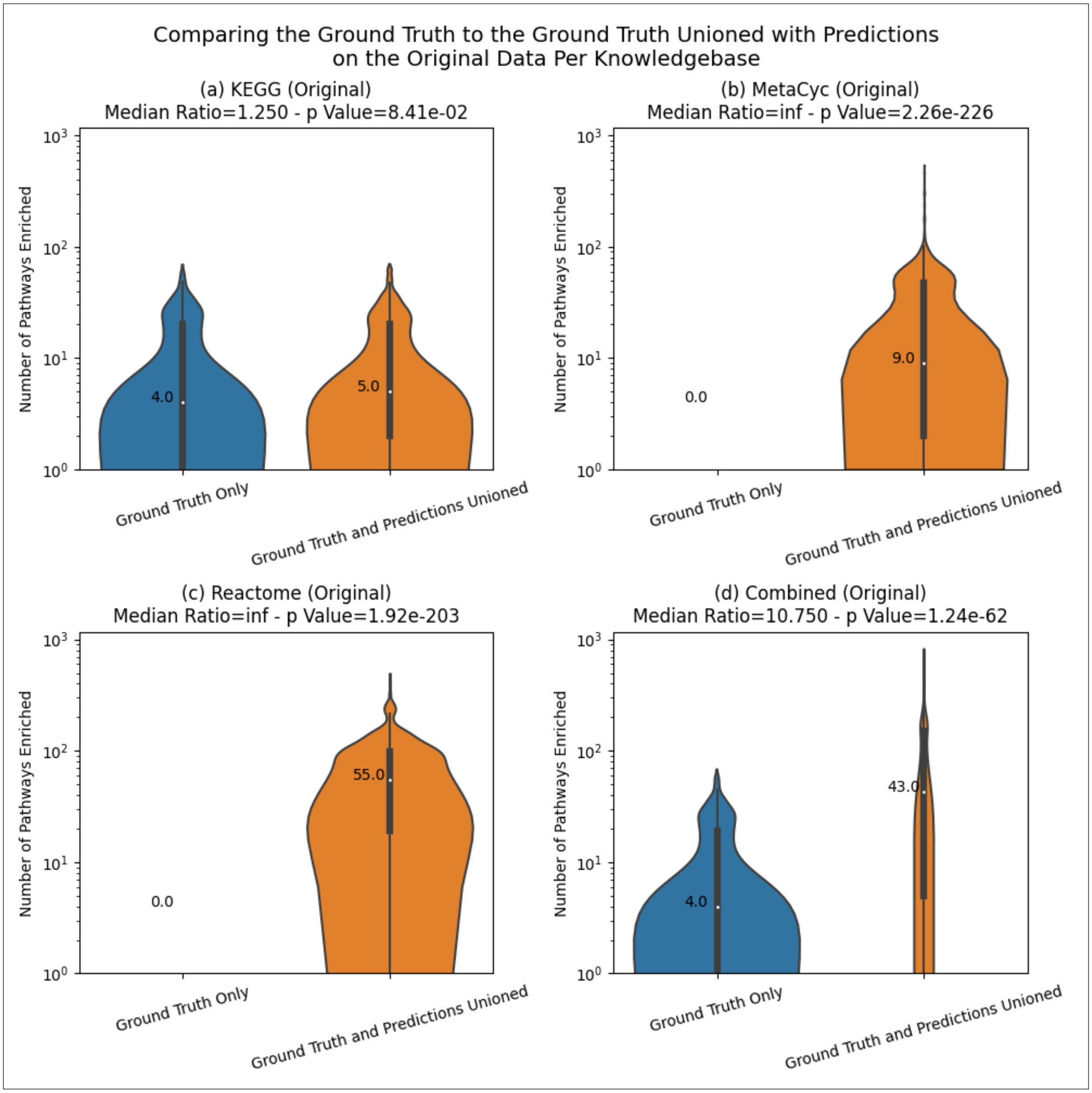

Figure S18 - Comparing the distribution across MW analyses of the number of enriched pathways between the ground truth pathway annotations and those unioned with the predicted annotations for the original MW datasets for each knowledgebase. Note that MetaCyc and Reactome had no ground truth annotations available since their metabolite IDs were not available in the original MW datasets. Combined results included for comparison. Results are for unique pathway definitions.

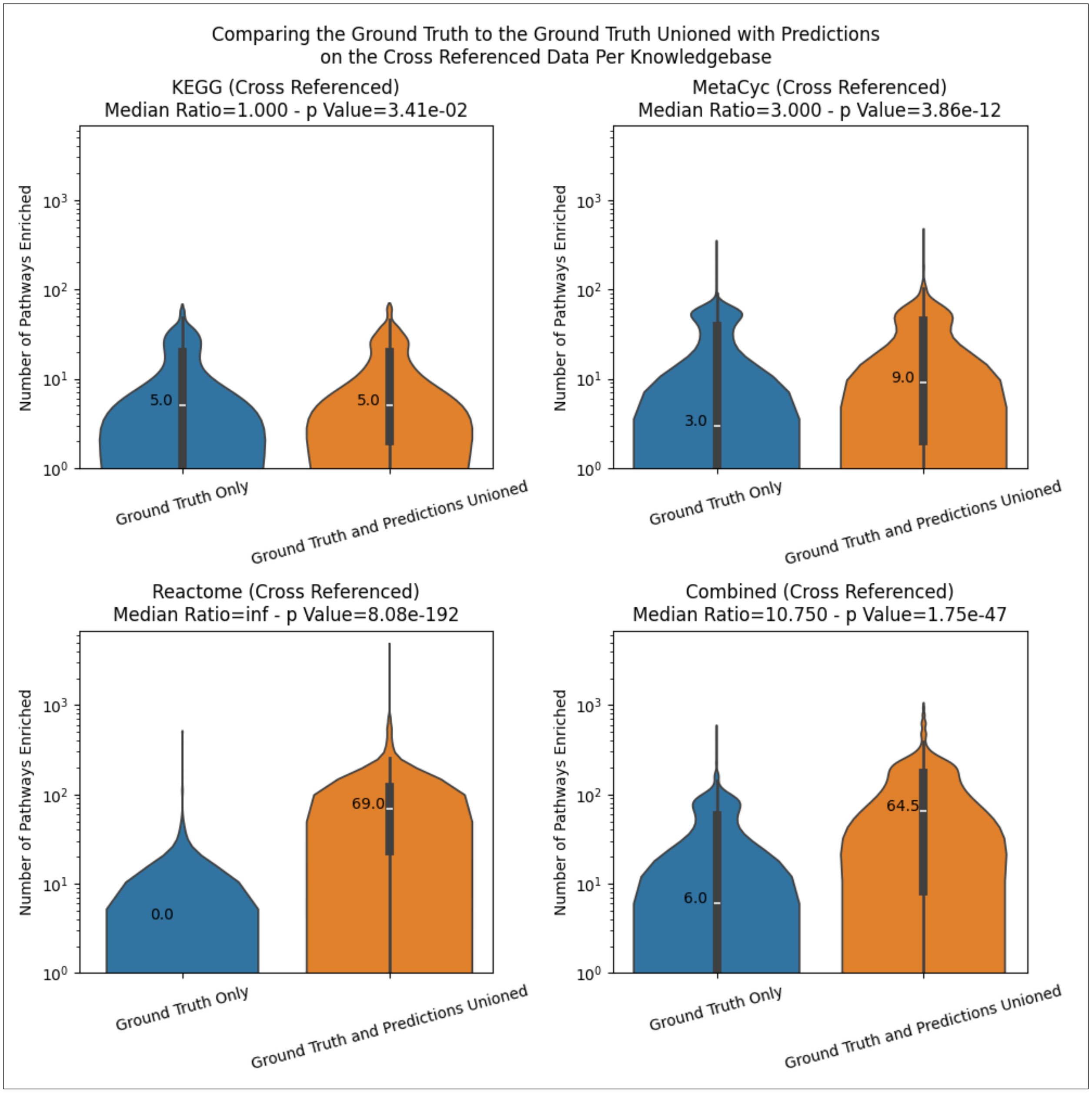

Figure S19 – Same as Figure S17 but for individual pathway IDs.

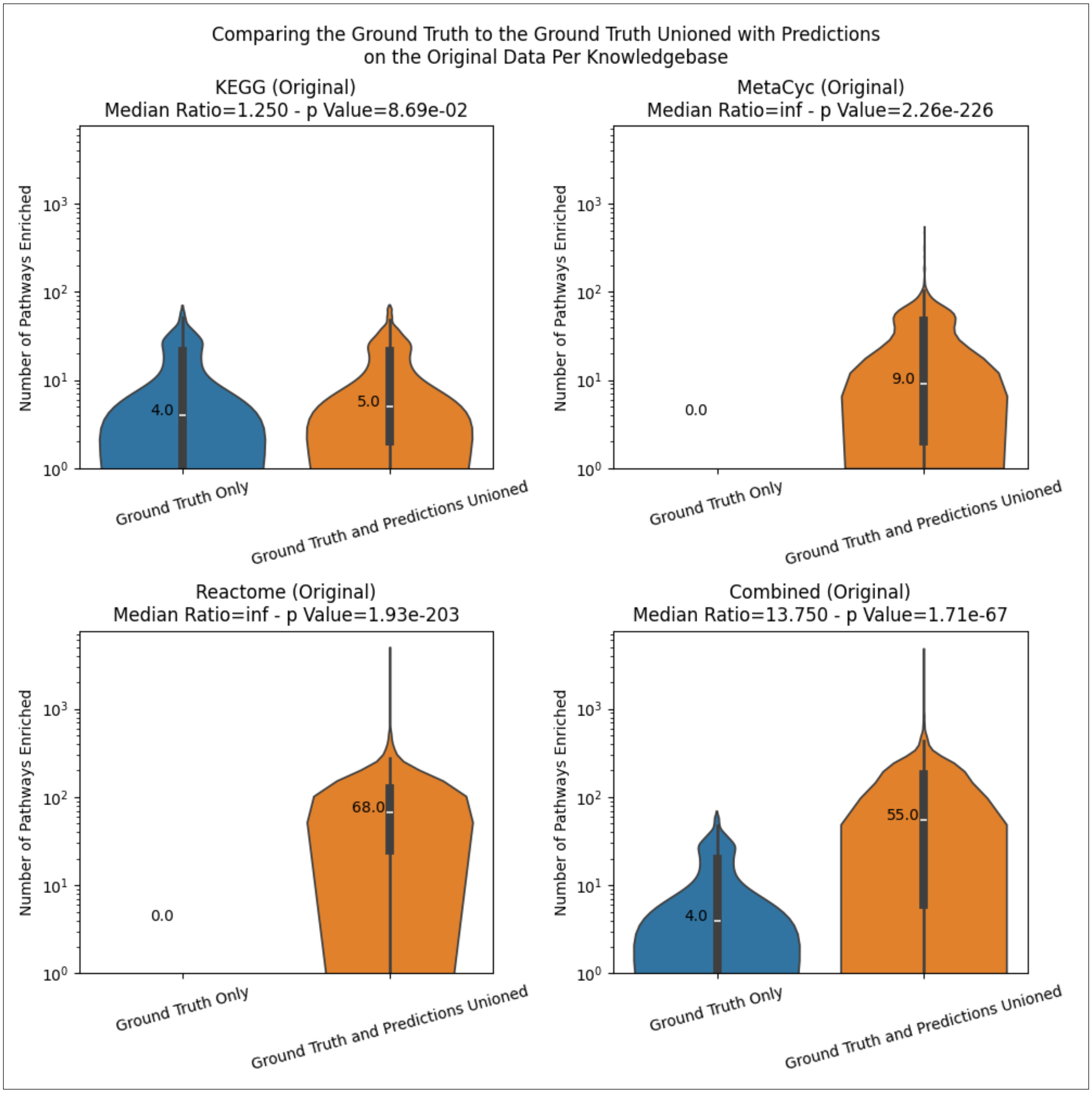

Figure S20 – Same as Figure S18 but for individual pathways.

Information Loss and Gain

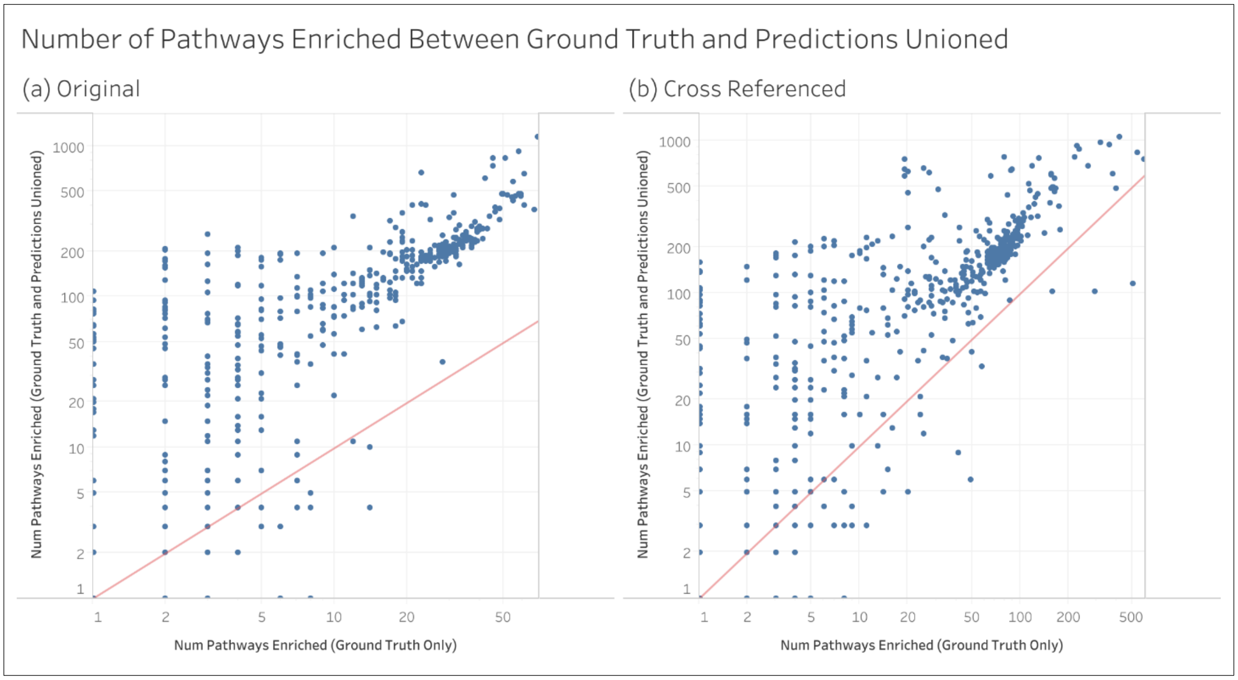

Figure S21 - The per dataset change in the number of enriched pathways between the ground truth and the ground truth unioned with the predictions. Each point is a separate MW dataset. Results are for individual pathway IDs

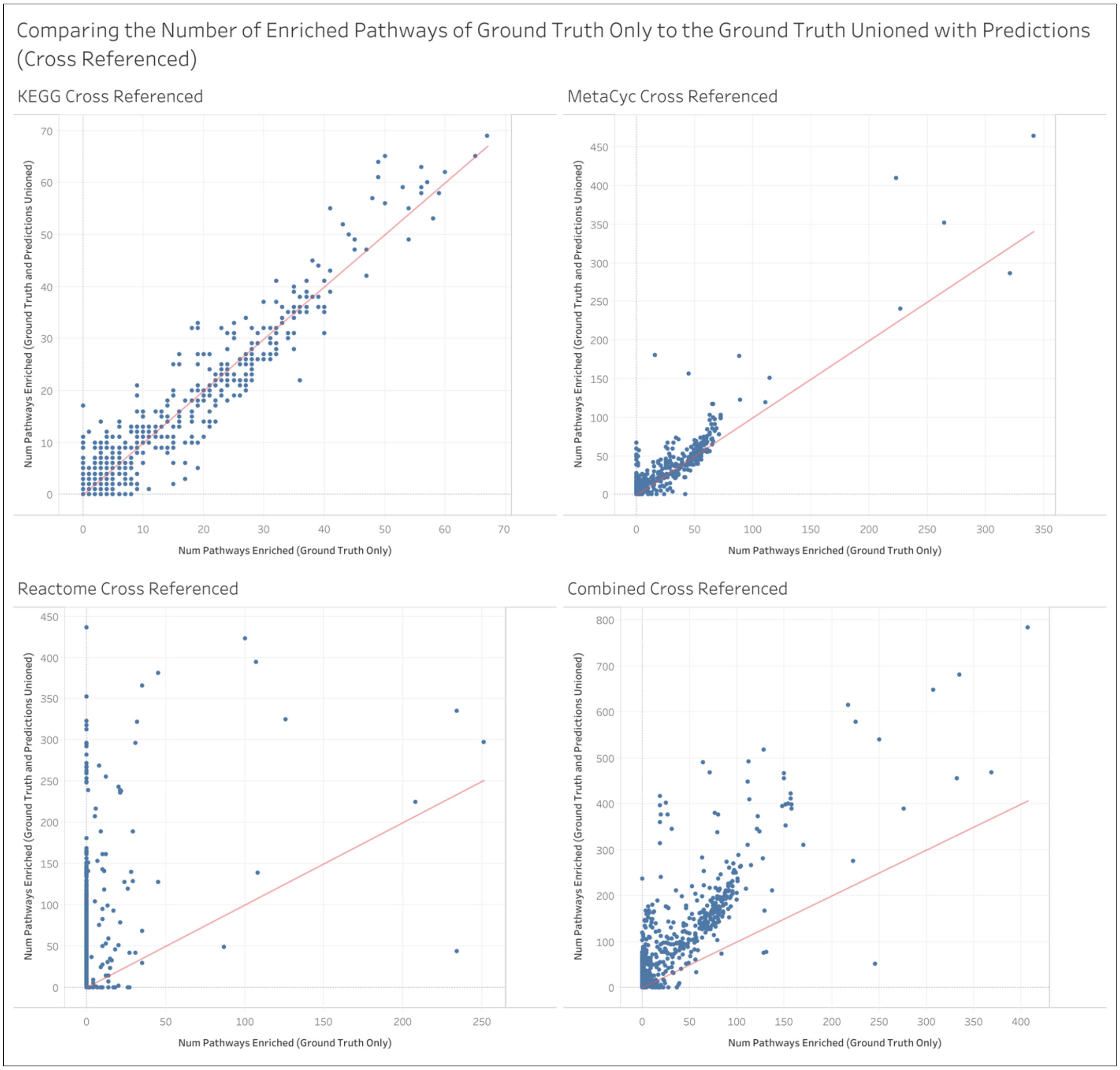

Figure S22 - Per analysis view of the change in the number of enriched pathways between the ground truth pathway annotations and those unioned with the predicted annotations for each knowledgebase on the cross-referenced MW datasets. Results are for unique pathway definitions.

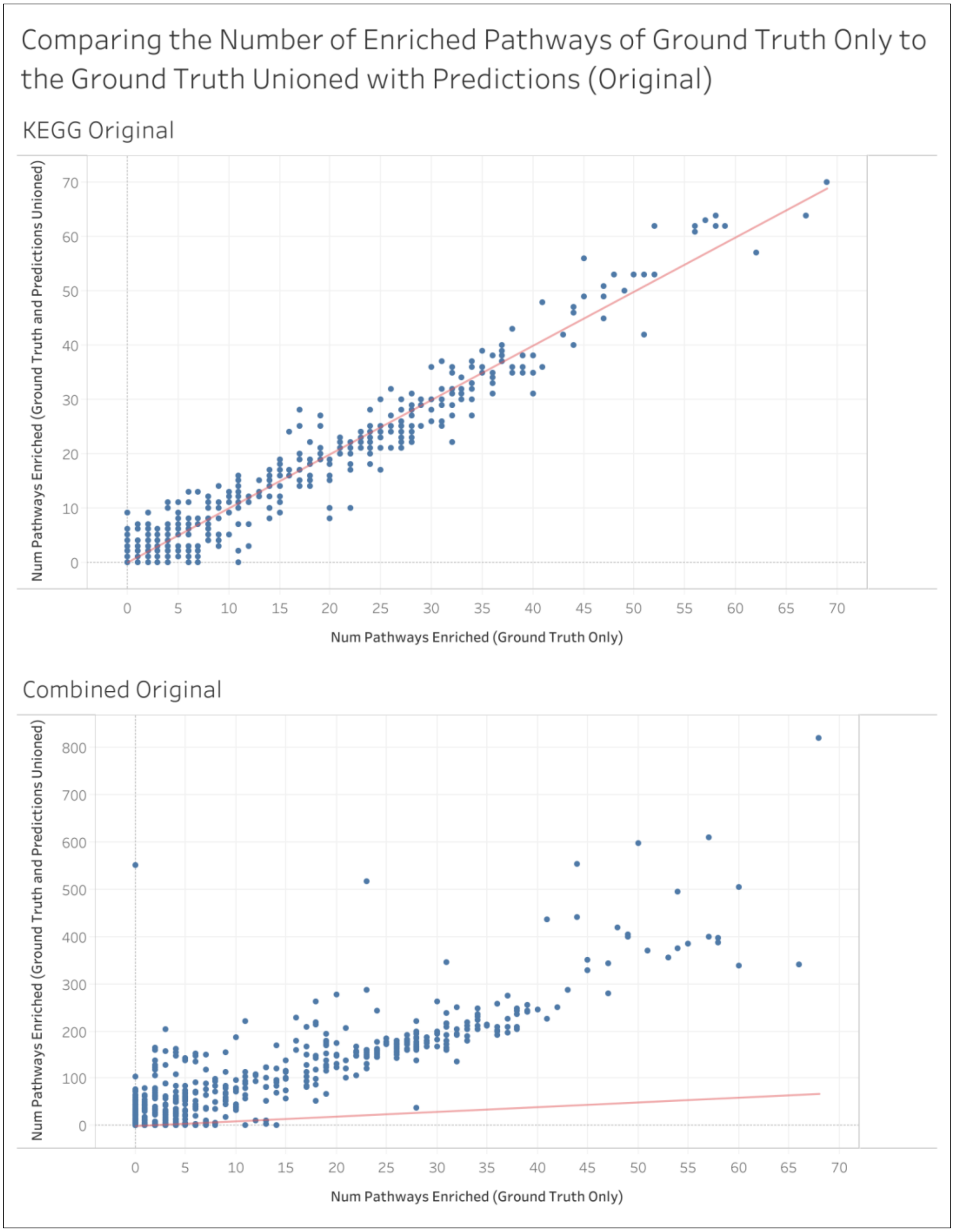

Figure S23 - Per analysis view of the change in the number of enriched pathways between the ground truth pathway annotations and those unioned with the predicted annotations for each knowledgebase on the original MW datasets. Note that MetaCyc and Reactome are not shown because these had no metabolite IDs available in the original datasets.

Results are for unique pathway definitions.

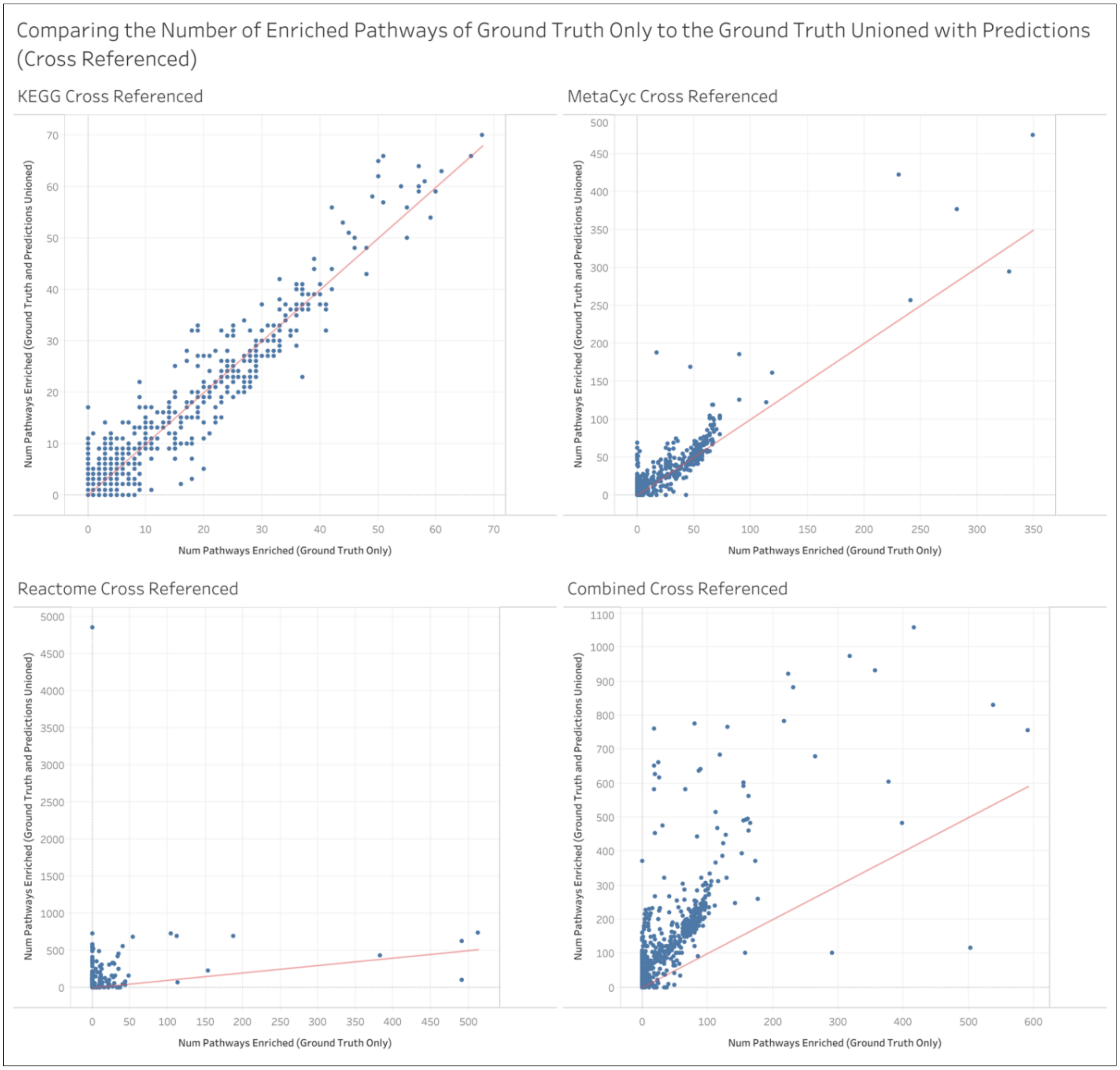

Figure S24 – Same as Figure S22 but for individual pathway IDs.

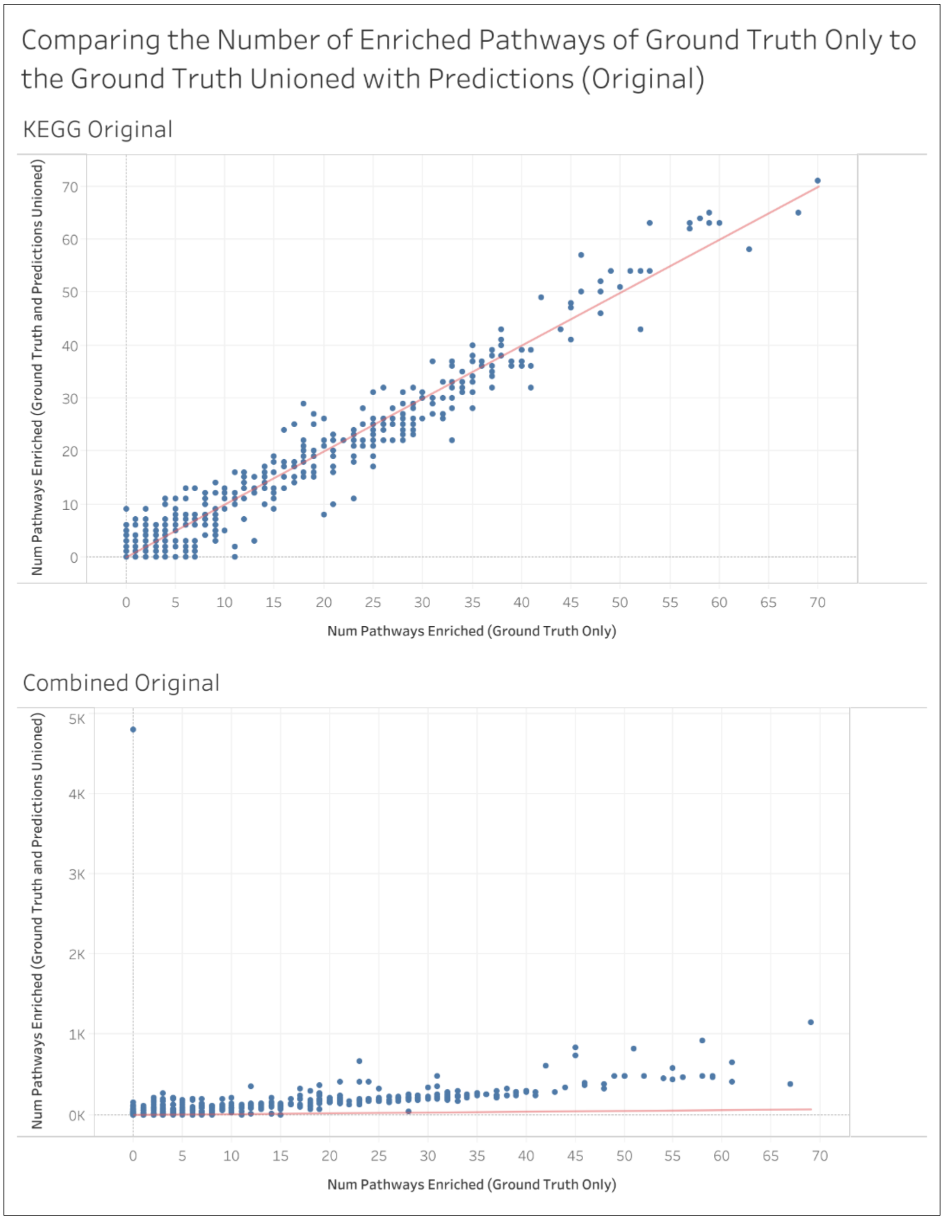

Figure S25 - Same as Figure S23 but for individual pathway IDs.

Table S2 -The amount of information gain and information loss summed across all enriched pathways and MW datasets. Results are for individual pathway IDs.

Analysis Type	Annotation Type	Num Ground Truth Detected Only	Num Predictions Detected Only	Num Both Detected	Total Detected	Num Lost %	Num Gained %	Unchanged %
Cross Referenced	Ground Truth and Predictions Unioned	4,137	65,032	22,084	91,253	4.5	71.3	24.2
	Predictions Only	4,080	69,234	22,141	95,455	4.3	72.5	23.2
Original	Ground Truth and Predictions Unioned	1,040	69,082	6,709	76,831	1.4	89.9	8.7
	Predictions Only	1,030	69,204	6,719	76,953	1.3	89.9	8.7

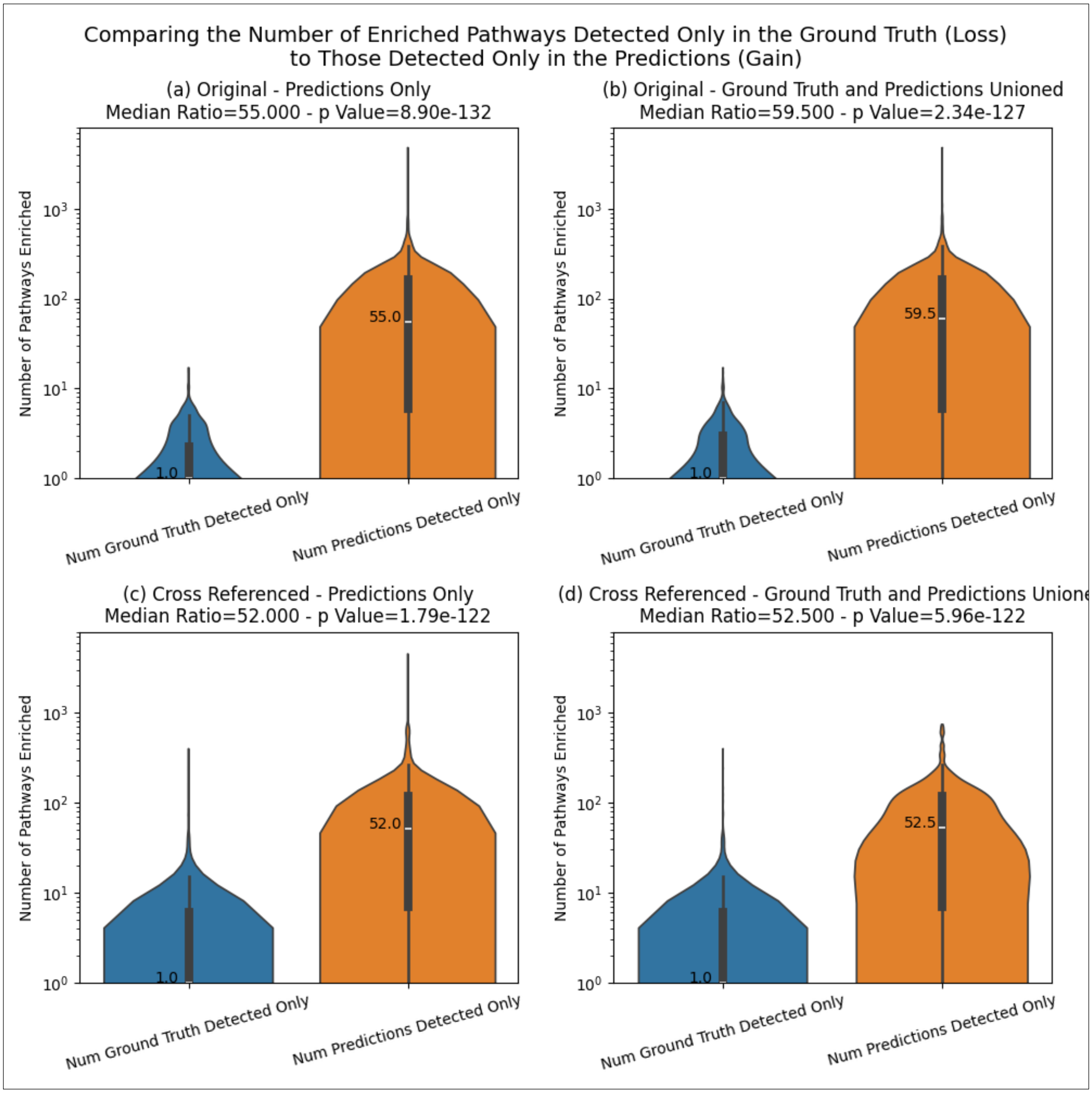

Figure S26 - Comparing the distribution of information loss to that of information gain across MW datasets. Results are for individual pathway IDs.

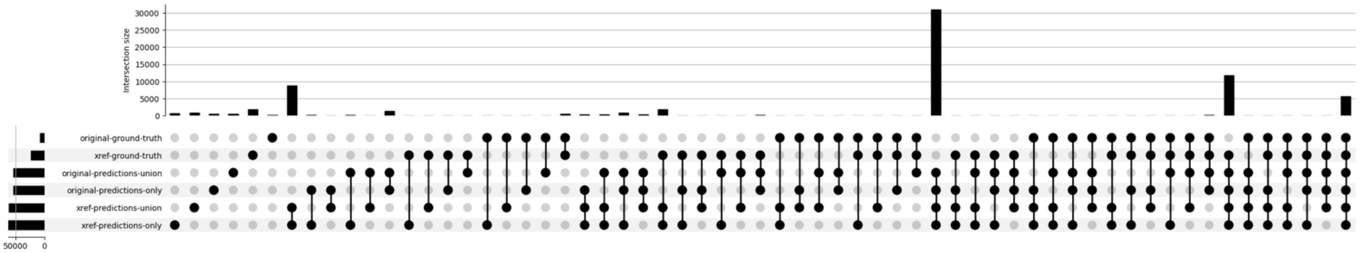

Figure S27 - Represents the information loss and information gain of enriched pathways between ground truth and predictions by showing the overlap of detected pathways. Results are for unique pathway definitions.

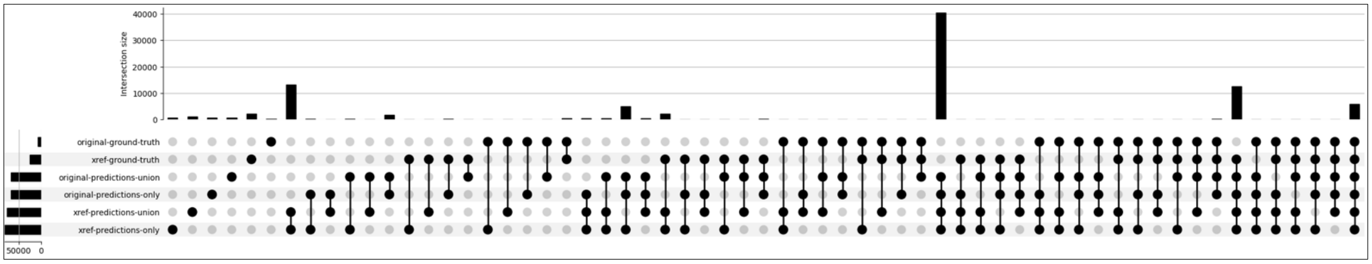

Figure S28 – Same as Figure S27 but for individual pathway IDs.

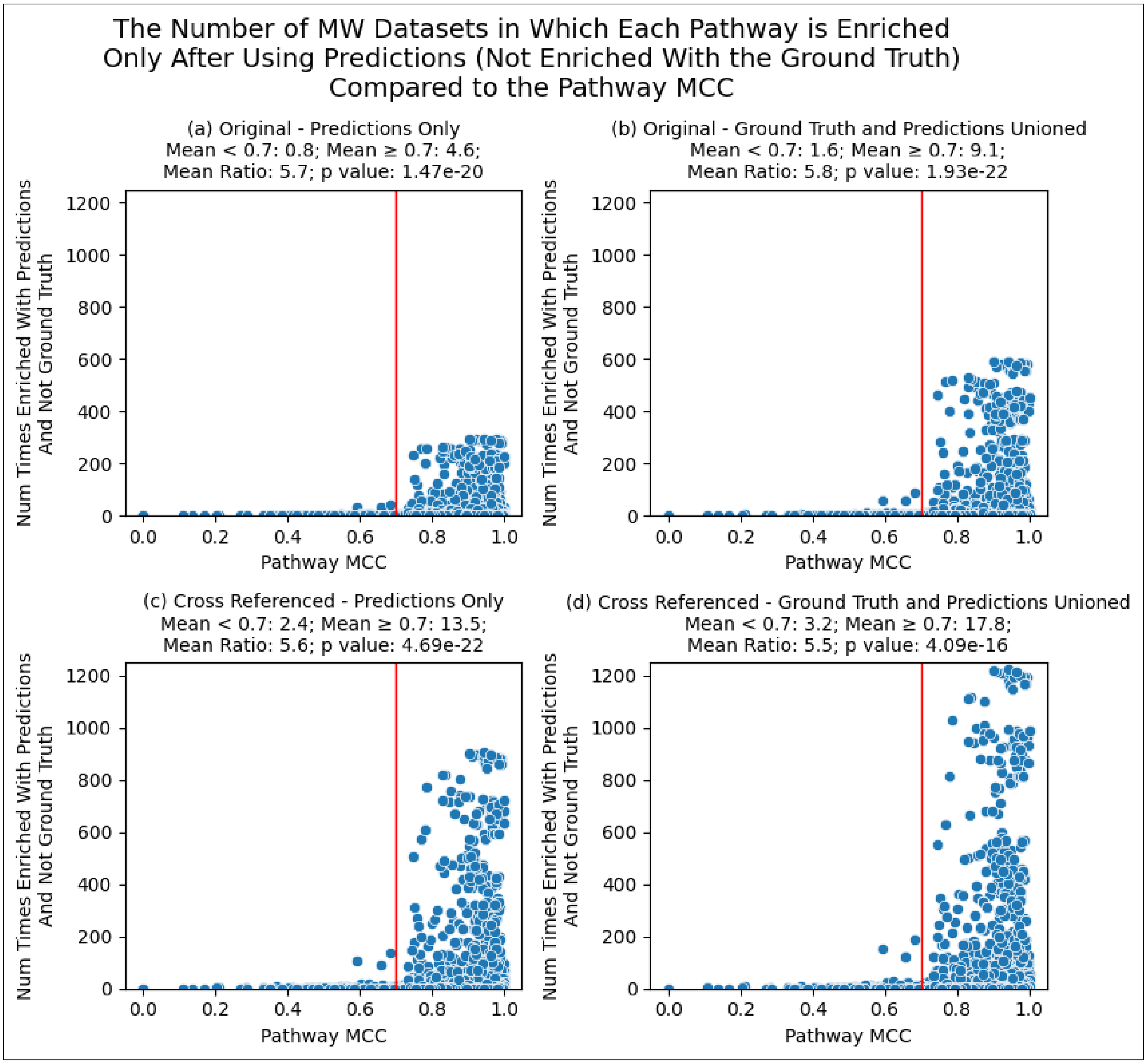

Figure S29 - Comparing the pathway MCC to the number of MW datasets wherein each pathway was not enriched when using ground truth annotations but was enriched when using predicted annotations. The red line is drawn at overall MCC = 0.7. Each point is a different pathway enriched in at least one dataset. Results are for individual pathway IDs.

Table S3 – The number of unique metabolites as part of metabolite-pathway annotations across MW datasets.

Analysis Type	Annotation Type	Mean	Median	Max	#Datasets
Original	Ground Truth	1919.1	0.0	6501	990
Original	Predictions Unioned	5102.1	0.0	18,411	990
Cross Referenced	Ground Truth	4905.4	0.0	18,141	990
Cross Referenced	Predictions Unioned	5241.3	228.0	18,424	990

Table S4 – Sum of 1 minus average Jaccard index between enriched pathway definitions and every other pathway definition.

Analysis Type	Annotation Type	Mean	Median	Max	#Datasets
Original	Ground Truth	6.8	0.0	64.3	990
Original	Predictions Unioned	63.4	0.0	2574.5	990
Cross Referenced	Ground Truth	23.7	0.0	530.9	990
Cross Referenced	Predictions Unioned	73.6	1.0	1002.9	990

Table S5 - Hyperparameters used for the ML model.

Activation Function	Hidden Layer Size	Num Layers	Dropout	Learning Rate	Beta 1	Beta 2	EPS	Weight Decay	Prediction Threshold
ReLU6	1229	3	0.063739	0.000719	0.565180	0.497934	0.000817	2.698780e-06	0.678975

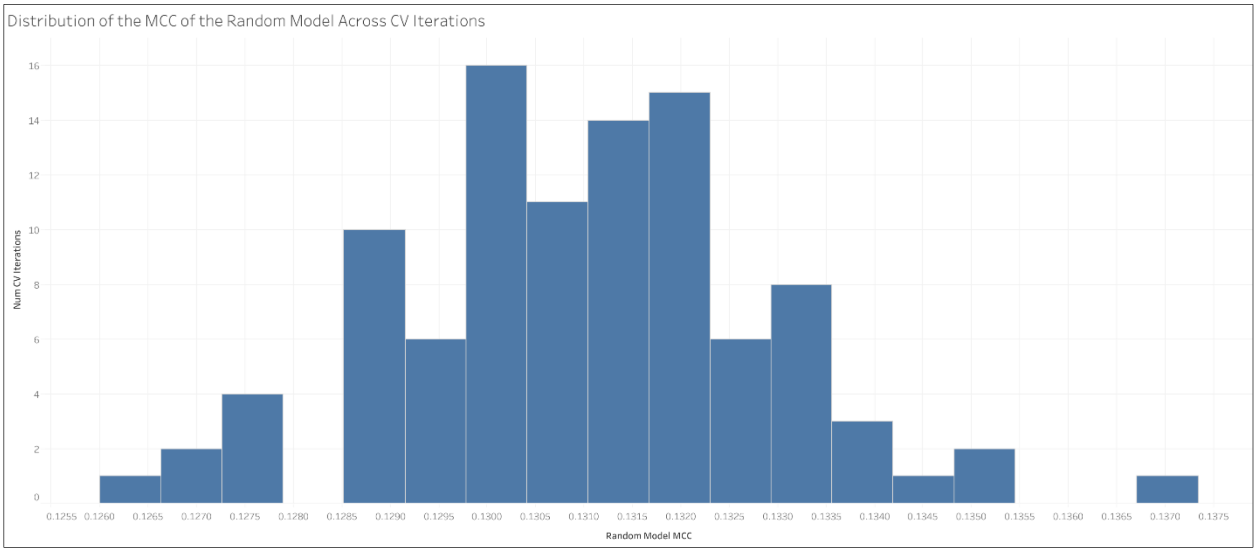

Figure S30 - Distribution of the MCC across 100 CV iterations, evaluating the performance of the random prediction model's ability to predict metabolite-pathway annotations.
